# Supplementary material for: Clinical communication in inflammatory bowel disease: a systematic review of the study of clinician–patient dialogue to inform research and practice
Source: BMJ Open. 2021 Aug 27;11(8):e051053. doi: 10.1136/bmjopen-2021-051053 (PMC8404434; doi:10.1136/bmjopen-2021-051053)
Supplement: Supplementary data [file bmjopen-2021-051053supp001.pdf]

Supplementary Table 1. Data items included in the data extraction template

- **Participant characteristics**
  - Participants and numbers
  - Participant socio-demographics
  - Additional health status information (e.g. pregnancy status, adult versus adolescent)
- **Disease characteristics**
  - Type of IBD
  - Disease activity
  - Disease phenotype
  - Extraintestinal manifestations
- **Study characteristics**
  - Year of research
  - Country of research
  - Research setting and type of consultation
  - Stated aims
  - Aims relevant to the review
  - Study design
  - Outcomes and measures
  - Stated findings
  - Stated conclusions
- **Consultation data and analysis characteristics**
  - Corpus size and number of consultations/episodes analysed in the study
  - Average length of consultations
  - One-off consultation or series
  - Data type (audio or video)
  - Method of linguistic data analysis (sociolinguistics, conversation analysis, content analysis, etc.)
  - Linguistic component/s analysed

Supplementary Table 2A. Analyses of risk of bias (tailored framework)

	Credibility									Reliability	
**Article**	**Type of consultation data**	**Size of consultation data analysed**	**Sample size justification**	**Linguistic component(s) analysed**	**Linguistic data analysis tool(s)**	**Demographic data**	**Additional contextual information**		**Arguments/findings supported by textual data (1-3: 1=NO and 3 =YES)**	**Unit of analysis**	**Second coder**
(20)	Nurse-patient consultation	95 video-recorded consultations (including patients who searched for medication-related online information prior to the consultation)	No	Lexis	Presence/absence of a list of words and the speaker	Yes	No		1	No	No
(21)	Nurse-patient consultation	59 video-recorded, 9 audio-recorded consultations (68 consultations)	No	Topic	An observation checklist (presence/absence)	Yes	No		1	No	Yes
(22)	Nurse-patient consultation	58 video- or audio-recorded consultations	No	Speech act	A data-driven codebook	Yes*	No		2	No	Yes
(23)	Gastroenterologist-patient consultation	68 audio- and video-recorded consultations	No	Lexis, Topic, Question asking, Contribution to conversation, Interruptions, framing	<u>Lexis:</u> presence/absence, context and co-text, speaker<br><u>Topic:</u> presence/absence, speaker<br><u>Question-asking:</u> open-ended vs. closed<br><u>Contribution to conversation:</u> Percentage of words spoken by the participants and the length of each visit<br><u>Interruptions:</u> Number<br><u>Framing:</u> Position in treatment sequence	Yes	Yes	• Patient and consultant interviews	2	No	No

(24)	Gastroenterologist-patient consultation	2 video-recorded consultation	No	Sitting, Reference to notes, Representation of space and time	*Proto-story*	No	Yes	• Patient interviews<br>• Information on patients' personal life and consultant level of experience	2	No	No

* Inconsistent reports of data on IBD type: While the total sample size was reported to be 58, information on IBD type was reported for 56 participants (34 participants with Crohn's disease, 20 participants with ulcerative colitis, and two participants with unknown disease).

Supplementary Table 2B. Analysis of risk of bias (Mixed Methods Appraisal Tool)

	**Mixed Methods Appraisal Tool (MMAT) – Version 2011**	**(20)**	**(21)**	**(22)**
Quantitative nonrandomized	Are participants (organizations) recruited in a way that minimizes selection bias?	No - 63.12% of participants had CD, 77.5% of the participants were moderately or highly educated	No - 62.3% of participants were female, 79.4% had CD, 73.5% were moderately or highly educated, 91.2% were Dutch	No – 35% of participants had UC, 87% of the participants were moderately or highly educated
	Are measurements appropriate (clear origin, or validity known, or standard instrument; and absence of contamination between groups when appropriate) regarding the exposure/intervention and outcomes?	Online Medical Information Seeking Behaviour: Patients explicitly asked if they used the internet to search for medical information ("yes" or "no") - **Yes**<br>Patient satisfaction: a 29-statement scale*– **Can't tell, tool could not be accessed**<br>Recall of medical information: a structured telephone interview using an adapted version of the Netherlands Patient Information Recall Questionnaire (NPIRQ)** - **Yes**<br>Medication adherence: A single item self-report measure *** - **Yes**	Immediate recall of medical information: An adapted version of The Netherlands Patient Information Recall Questionnaire (NPIRQ) - **Yes**<br>Delayed recall of medical information: An adapted version of the immediate recall questionnaire based on each individual consultation - **Yes (immediate and delayed recall measures were not the same)**<br>Percentage of accurate recall: The ratio of the number of the accurately recalled items to the total number of items discussed coded by two coders using videotaped visits - **Yes**<br>Total recall score: the mean recall percentage per patient for immediate and delayed recall - **Yes**<br>Medication intake behaviour: A single item self-report measure ("Please indicate on a scale from 1	Yes - Communication strategies to Discuss Online Health Information: A data-driven codebook

			to 10 the extent to which you are taking the medication as prescribed)" - **Yes**	
	In the groups being compared (exposed vs. non-exposed; with intervention vs. without; cases vs. controls), are the participants comparable, or do researchers take into account (control for) the difference between these groups?	N/A	N/A	N/A
	Are there complete outcome data (80% or above), and, when applicable, an acceptable response rate (60% or above), or an acceptable follow-up rate for cohort studies (depending on the duration of follow-up)?	Yes	Yes	Yes
Qualitative	Are the sources of qualitative data (archives, documents, informants, observations) relevant to address the research question (objective)?	Yes; However, only those consultations in which internet-related words were included in the analysis.	Yes	Yes; However, only those consultations in which internet-related words were included in the analysis. Consultations in which there was an implicit reference to online health information were not included.
	Is the process for analyzing qualitative data relevant to address the research question (objective)?	Yes	Yes	Yes
	Is appropriate consideration given to how findings relate to the context, e.g., the setting, in which the data were collected?	No - Discussion of online health information was regarded as a dichotomy. How the online information was discussed was not studied.	No - the effect of contextual factors was not considered in analysing the content of consultations.	No – While the co-text of communicative strategies was discussed to some degree, the effect of contextual factors was not considered in the analysis.
	Is appropriate consideration given to how findings relate to researchers' influence, e.g., through their interactions with participants?	Yes	The likely influence of video-recording consultation on participants' behaviour was not addressed.	Yes
Mixed methods	Is the mixed methods research design relevant to address the qualitative and quantitative research questions (or objectives), or the qualitative and quantitative aspects of the mixed methods question (or objective)?	Yes	Yes	Yes
	Is the integration of qualitative and quantitative data (or results) relevant to address the research question (objective)?	Yes	Yes	Yes

	Is appropriate consideration given to the limitations associated with this integration, e.g., the divergence of qualitative and quantitative data (or results) in a triangulation design?	N/A	N/A	N/A
Screening questions	Are there clear qualitative and quantitative research questions (or objectives*), or a clear mixed methods question (or objective)?	Yes	Yes	Yes
	Do the collected data allow address the research question (objective)? E.g., consider whether the follow-up period is long enough for the outcome to occur (for longitudinal studies or study components).	Yes	Yes	Yes

CD, Crohn’s disease; UC, ulcerative colitis

* Linn AJ, van Weert JCM, van Dijk L, Horne R, Smit EG. The value of nurses’ tailored communication when discussing medicines: Exploring the relationship between satisfaction, beliefs and adherence. Journal of Health Psychology. 2016;21(5):798-807

** Jansen J, van Weert J, van der Meulen N, van Dulmen S, Heeren T, Bensing J. Recall in older cancer patients: measuring memory for medical information. Gerontologist. 2008;48(2):149-57.

*** Linn AJ, van Dijk L, Smit EG, et al. May you never forget what is worth remembering: the relation between recall of medical information and medication adherence in patients with inflammatory bowel disease. J Crohns Colitis 2013;7:e543–50.
