# Supplementary material for: Clinical communication in inflammatory bowel disease: a systematic review of the study of clinician–patient dialogue to inform research and practice
Source: BMJ Open. 2021 Aug 27;11(8):e051053. doi: 10.1136/bmjopen-2021-051053 (PMC8404434; doi:10.1136/bmjopen-2021-051053)
Supplement: Author's manuscript [file bmjopen-2021-051053.draft_revisions.pdf]

BMJ Open is committed to open peer review. As part of this commitment we make the peer review history of every article we publish publicly available.

When an article is published we post the peer reviewers' comments and the authors' responses online. We also post the versions of the paper that were used during peer review. These are the versions that the peer review comments apply to.

The versions of the paper that follow are the versions that were submitted during the peer review process. They are not the versions of record or the final published versions. They should not be cited or distributed as the published version of this manuscript.

BMJ Open is an open access journal and the full, final, typeset and author-corrected version of record of the manuscript is available on our site with no access controls, subscription charges or pay-per-view fees (http://bmjopen.bmj.com).

If you have any questions on BMJ Open's open peer review process please email info.bmjopen@bmj.com

BMJ Open

Clinical communication in inflammatory bowel disease: a systematic review of the study of clinician-patient dialogue to inform research and practice

Journal:	*BMJ Open*
Manuscript ID	bmjopen-2021-051053
Article Type:	Original research
Date Submitted by the Author:	09-Mar-2021
Complete List of Authors:	Karimi, Neda; Ingham Institute; University of New South Wales, South Western Sydney Clinical School<br>Kanazaki, Ria; Liverpool Hospital, Gastroenterology Department; University of New South Wales, Ingham Institute of Applied Medical Research<br>Lukin, Annabelle; Macquarie University, Department of Linguistics, Faculty of Medicine, Health and Human Sciences, Macquarie University<br>Moore, Alison; University of Wollongong Faculty of Law Humanities and the Arts<br>Williams, Astrid-Jane; Liverpool Hospital, Department of Gastroenterology; Ingham Institute,<br>Connor, Susan; University of New South Wales, South Western Sydney Clinical School; Liverpool Hospital, Department of Gastroenterology
Keywords:	Inflammatory bowel disease < GASTROENTEROLOGY, QUALITATIVE RESEARCH, SOCIAL MEDICINE

SCHOLARONE™
Manuscripts

I, the Submitting Author has the right to grant and does grant on behalf of all authors of the Work (as defined in the below author licence), an exclusive licence and/or a non-exclusive licence for contributions from authors who are: i) UK Crown employees; ii) where BMJ has agreed a CC-BY licence shall apply, and/or iii) in accordance with the terms applicable for US Federal Government officers or employees acting as part of their official duties; on a worldwide, perpetual, irrevocable, royalty-free basis to BMJ Publishing Group Ltd ("BMJ") its licensees and where the relevant Journal is co-owned by BMJ to the co-owners of the Journal, to publish the Work in this journal and any other BMJ products and to exploit all rights, as set out in our licence.

The Submitting Author accepts and understands that any supply made under these terms is made by BMJ to the Submitting Author unless you are acting as an employee on behalf of your employer or a postgraduate student of an affiliated institution which is paying any applicable article publishing charge ("APC") for Open Access articles. Where the Submitting Author wishes to make the Work available on an Open Access basis (and intends to pay the relevant APC), the terms of reuse of such Open Access shall be governed by a Creative Commons licence – details of these licences and which Creative Commons licence will apply to this Work are set out in our licence referred to above.

Other than as permitted in any relevant BMJ Author's Self Archiving Policies, I confirm this Work has not been accepted for publication elsewhere, is not being considered for publication elsewhere and does not duplicate material already published. I confirm all authors consent to publication of this Work and authorise the granting of this licence.

For peer review only

Clinical communication in inflammatory bowel disease: a systematic review of the study of clinician-patient dialogue to inform research and practice

Neda Karimi[1,3], PhD; Ria Kanazaki[1,2,3], MD; Annabelle Lukin[4], PhD; Alison Rotha Moore[5], PhD; Astrid-Jane Williams[1,2], MD; Susan J Connor[1,2,3], PhD

[1]Ingham Institute for Applied Medical Research, Sydney, Australia
[2]Department of Gastroenterology, Liverpool Hospital, Sydney, Australia
[3]South Western Sydney Clinical School, The University of NSW, Sydney, Australia
[4]Department of Linguistics, Faculty of Medicine, Health and Human Sciences, Macquarie University, Sydney, Australia
[5]School of Humanities and Social Inquiry, Faculty of Law, Humanities and the Arts, The university of Wollongong, Wollongong, Australia

Corresponding author: Neda Karimi, PhD
Postal address: Gastroenterology Research Group, Ingham Institute, Liverpool Hospital, Locked Bag 7103, LIVERPOOL BC NSW 1871 Australia
Email Address: neda.karimi@unsw.edu.au
Phone: +61 (02) 8738 9247

For peer review only

ABSTRACT

Objectives: This systematic review aims to investigate what is currently known about the characteristics of interactions between patients with inflammatory bowel disease (IBD) and their clinicians and its effect on patient outcomes.

Method: Seven databases were systematically searched. Journal articles and book chapters in English published up to May 2020 were included. Title, abstract, and full text of identified articles were assessed by three reviewers. Data were extracted using a tailored data extraction template and the risk of bias was assessed using a specifically developed quality assessment tool, grounded in linguistic theory, as well as the Mixed Methods Appraisal Tool.

Results: Of the 2257 abstracts reviewed, 44 full articles were retrieved. Of these four met the inclusion criteria and thus formed the basis of the review. Interactions between IBD nurses and patients have been characterised in terms of information on prescribed medications without consideration of the interpersonal aspect. Discussing online medical information has been shown to improve patient satisfaction. Analyses of gastroenterologist-patient interactions have concentrated on the clinical relationship which has been shown to be disease-centred. Shared decision-making in ulcerative colitis has been shown to be compromised due to lack of transparency regarding treatment goals.

Conclusions: There is a paucity of research that examines the different aspects of clinical communication in IBD and their effect on patient outcomes.

Keywords: clinical encounter, clinical interaction, clinician-patient relationship, communication, Crohn’s disease, inflammatory bowel disease, ulcerative colitis

STRENGTHS AND LIMITATIONS OF THIS STUDY

- This systematic review summarises and interprets the available evidence on clinical communication in IBD by assessing evidence resulting from investigations of authentic clinical interactions in IBD (real-life data) rather than investigations of self-reported data (perceptions and attitudes).
- The review identifies a dearth of research on clinical communication in IBD and provides several recommendations for further research in this area.
- The review is based on four articles only and, therefore, does not provide a complete picture of clinical communication in IBD and its effect.

For peer review only

INTRODUCTION

The main space in which clinicians and patients manage care, negotiate roles, and make decisions is their interaction during consultations. Understanding communication between clinicians and patients in this space and its existing variations is crucial for understanding the bigger picture of how disease is managed.

In inflammatory bowel disease (IBD), clinical communication is argued to affect patient satisfaction, treatment adherence, patient quality of life, disease management, and self-management (1-4). In addition, in tandem with the recognition of the importance of minimising disease activity at an early stage in IBD - using a 'treat-to-target' (T2T) management approach (5, 6), research promoting shared decision-making in IBD has gained momentum (7). A T2T approach involves personalised care and early interventions aimed at delaying or preventing disease progression, preventing bowel damage, and promoting mucosal healing (6). It is a collaborative approach involving the physician (and the multidisciplinary team) and the patient (6). It involves joint risk-benefit assessment and decision-making, monitoring, and optimising therapy to achieve disease control and symptom improvement (5, 8). As a result, discourses around communication and the role of the patient as a key stakeholder in decision-making have gained recognition in IBD research (8).

Currently, studies that investigate communication between IBD clinicians and patients through investigation of the "clinician-patient interface" (9) - i.e. projects that investigate interactions between patients and clinicians, rather than patients' perceptions of clinical communication - are less common. The current review ascertains the existing knowledge in this area in order to inform the field, identify areas that require further investigation, and position this literature within current IBD care practice and research. The main objective is to identify, organise and summarise systematically what is currently known about (a) the characteristics of conversations between clinicians that manage IBD and patients with IBD, and (b) how clinical discussion affects health outcomes in IBD. A secondary objective is to identify the different approaches to the analysis of clinical interaction in IBD, the methodological trends, and the gaps for future research. Underpinning both of these objectives, it is also our aim to show the importance of studying the details of how clinical communication takes place in the specific context of IBD and why drawing only on highly generic principles of health care communication or research involving patients with other health conditions is not sufficient for maximising the delivery of quality care in IBD.

METHOD

Study selection, data extraction, quality assessment, and synthesis

The protocol for this systematic review is registered with PROSPERO (CRD42020169657) and separately published (10).

Published peer-reviewed journal articles and book chapters in English that investigated the characteristics of naturally occurring interactions between clinicians that manage IBD patients and patients with IBD during recorded consultations were included in this review. Self-report studies that explored clinicians' and patients' attitudes, or beliefs about clinical interaction only were excluded. Target participant groups included patients with IBD and clinicians that manage IBD patients in primary and secondary health care (e.g. general

For peer review only - http://bmjopen.bmj.com/site/about/guidelines.xhtml

practitioners, IBD specialists, IBD nurses), complementary and alternative medicine (e.g. acupuncturists, traditional Chinese medicine practitioners), or allied health (e.g. dietitians). Health care providers whose primary treatment includes the interaction itself (e.g. psychotherapists) were not included. Studies in which eligible participant groups were present but IBD was not the focus of the study were also excluded.

Scopus, PubMed, Embase, Communication Abstracts, Health & Society, Linguistics and Language Behavior Abstracts (LLBA), and PsycINFO were searched from inception to May 2020. The reference lists of eligible as well as excluded but relevant publications were screened. Subject matter experts were consulted to ensure inclusiveness. The search strategy is available in our published protocol (10).

Titles and abstracts were screened by three reviewers (NK, AL, and RK). The reviewers identified eligible publications based on their title and abstract, compared their lists of selected publications, and resolved any discrepancies prior to the full-text review. Full-text publications were screened for final inclusion by the reviewers with complete agreement between the reviewers.

Data were extracted from included articles by one reviewer (NK) and checked by the review team for accuracy. The template used for data extraction was developed in consultation with the existing health communication and linguistics literature including previous systematic literature reviews of this kind (9, 11-14) and included the data items presented in Supplementary Table 1.

Risk of bias was assessed using a template designed by the review team in consultation with previous systematic reviews of this kind (13) and compatible with the principles of systemic functional linguistics (15, 16) which offers a conceptual framework for the architecture of language, tools to measure the different components of language, and methodological approaches for principled selection of data and data analysis tools to reduce the description bias and increase credibility. For more information on the assessment of risk of bias refer to the protocol paper (10). In addition to the tailored template, to assess risk of bias in mixed-methods studies in which the analysis of talk was a secondary aim, the Mixed Methods Appraisal Tool (MMAT) (17) was used.

The extracted data were summarised and compared in overview tables and figures and are interpreted in a narrative synthesis. Halliday's concept of metafunction was used to classify the communicative aspects analysed by the included studies. The concept of metafunction is derived from the view that language has evolved in and through the social contexts in which it functions, and this contextual pressure in its evolution produced three main organising principles for language. All languages, despite their considerable variation, give their speakers resources for (1) construing human experiences (ideational metafunction), (2) enacting personal and social relationships (interpersonal metafunction), and (3) organising a discursive flow and creating cohesion and continuity (textual metafunction) (18). Each linguistic feature analysed by the included papers was coded depending on which of these functions it served. Classifying the included publications based on the metafunction addressed has the advantage of identifying systematically the aspects of IBD clinical communication which have been described as well as those that are not yet explored.

For peer review only - http://bmjopen.bmj.com/site/about/guidelines.xhtml

Ethical statement

Ethics reviews and approval were not required for this systematic review as no primary data were collected.

Patient and public involvement

Patients or the public were not involved in the design and conduct of this systematic review.

RESULTS

Study selection and study characteristics

Of the 2257 studies identified through systematic searching, four papers from four unique studies fulfilled the inclusion criteria. Figure 1 illustrates the screening and inclusion process.

Insert Figure 1. Here (19)

Included studies were conducted in six countries (The Netherlands, UK, Italy, Germany, France, and USA) and in two institutional contexts (outpatient IBD nurse consultation, outpatient specialist consultation). Studies were published between 2008 and 2018. The characteristics of included studies are described in Table 1 and the characteristics of the interaction data used in these studies are summarised in Table 2.

Risk of bias within studies

Following assessment of risk of bias (Supplementary Tables 2a and 2b), all four publications were included in data synthesis.

Two publications (20, 21) were mixed-methods correlational studies mainly examining selected predictive variables and their relationship with medication adherence. The aim in these projects was to identify the predictors of medication intake behaviour which was facilitated through investigation of nurse-patient interaction. The analysis of the interactions was limited to determining the presence or absence of preselected pieces of information. The analysis of clinical talk by Sanders and Linn (20) was limited to coding the interaction in terms of presence of the words *internet*, *Google(d)*, *webpages*, *fora*, *online*, or any other internet-related words and the person initiating such discussion. Similarly, Linn and colleagues' (21) analysis included coding the data in terms of the presence or absence of certain pieces of information regarding prescribed medication. Other than the demographic data, no contextual information was collected in the two publications. Nor were the analyses accompanied by examples of interactions. However, since the collected data and the processes for analysing qualitative data were relevant to address the research questions (refer to MMAT analysis), the two publications were included in the review.

The other two publications (22, 23) were descriptive studies and more detailed in terms of their linguistic analysis. Both studies combined analysis of recorded IBD specialist consultations with patient interviews. Rubin and colleagues (22) conducted physician interviews, in addition to patient interviews, and investigated physician-patient alignment. The studies provided examples from their textual data to support the findings, however this was confined to single conversational turns or excerpts from single turns in Rubin et al (22). In contrast, Radley and colleagues (23) included extra contextual data such as the consultant's level of experience and information on the personal life of the patient in their

For peer review only - http://bmjopen.bmj.com/site/about/guidelines.xhtml

analysis, and included evidence from excerpts of gastroenterologist-patient interactions that illustrate patterns such as turn-taking and how topics are initiated and responded. However, these excerpts were taken from three consultations only.

None of the four studies provided sample size justification or mentioned use of a unit of analysis in their analysis. Only Linn and colleagues determined interobserver reliability for text coding.

Results: Nurse-patient communication

Studies investigating nurse-patient communication in this review focused on the effect of communicating information regarding prescribed medicines (ideational metafunction) on patient medication intake behaviour. No study has looked at the dynamics of the nurse-patient relationship (interpersonal metafunction), the structure of nurse-led consultations or the continuity of dialogue and how subjects introduced by patients are maintained or ignored (textual metafunction).

Figure 2 summarises the findings of those publications in terms of the characteristics of nurse-patient interactions and their effects on patient outcomes and categorises these findings in terms of the linguistic function they address.

Insert Figure 2 Here

Results: Gastroenterologist-patient communication

Studies that investigated gastroenterologist-patient communication were different from those that investigated nurse-patient communication in their focus and objectives. The studies mainly focused on the interpersonal relationship between the gastroenterologist and the patient and the treatment decision-making process with the aim to reduce the distance between the patient and the consultant.

Figure 3 summarises the findings of the two articles that described the characteristics of the gastroenterologist-patient communication and categorises these findings in terms of the linguistic function they investigate.

Insert Figure 3 Here

INTERPRETATION

Nurses as transmitters of information

The IBD health communication literature identifies nurses only as transmitters of information about new medications. Assuming nurse-patient interactions to have only an ideational function is arguably a simplifying practice. For example, using this methodology, data relating to turn-taking, boundaries (who initiates a topic and who closes a topic), evaluation and attitudes, elaboration, or engagement are not captured. The interpersonal function of the nurse-patient communication in IBD has more recently started to attract the attention of IBD researchers. In a 2020 study conducted by Linn and colleagues in the Netherlands and published after May 2020 (outside the scope of this review) (24), the way online health information-seeking is discussed by patients and nurse practitioners during prescription consultations has been investigated from an interpersonal perspective.

For peer review only - http://bmjopen.bmj.com/site/about/guidelines.xhtml

Nurses are at the forefront of IBD healthcare, providing medical and psycho-social care (including but not limited to provision of information) via IBD advicelines and virtual clinics and are at times the mediator or gatekeeper between the patient and the gastroenterologist (25-30). Understanding the dynamics of the IBD nurse-patient interaction as a bidirectional process that both responds to the context at hand and reshapes it will help develop effective communication strategies and identify communicative risks. In achieving this understanding, a robust account of variation in nurse-patient interaction is crucial since small changes in ways of speaking can produce big differences in how a context is experienced and interpreted by patients. The current literature, seemed to use 'common sense' rather than evidence, to provide nurse-patient communication recommendations. For example, Linn et al.'s (21) suggestion that signposting and categorising information can improve recall was not grounded in the findings of their study. These recommendations seem to be hypotheses that would need testing.

Discussion of online health information

Sanders and Linn showed that only about half the patients who had searched for medical-related online information prior to the nurse prescription consultation discussed or had the opportunity to discuss that information (20). Barriers to discussing online health information are known from studies outside IBD to include clinician's resistance towards discussing such information, patient concerns surrounding disapproval by the clinician or fear of embarrassment (31). It has also been shown outside IBD that men are more likely than women to have a conversation regarding online information with physicians (32). Internet health information seeking and discussing such information during the consultation can empower patients and give them a sense of control and confidence when talking to their clinician (31). It can also help them gain greater clarity, orientation, and certainty regarding their own health and lead to higher patient satisfaction as shown in this review, so it seems important to increase opportunities for such discussion. Research has shown that the clinician's communication skills and their open response to a less hierarchised relationship as a result of a patient discussing online health information is important in forming a positive relationship with the patient (31).

Gastroenterologist-patient communication

Gastroenterologist-patient communication has been conceptualised around the interpersonal relationship between the gastroenterologist and the patient and its role in the decision-making process. However, the focus of the literature is on the gastroenterologist's contribution to the dialogue with less emphasis on contributions from the patient. Furthermore, little information is provided on the structure of the IBD specialist consultations. What is known, in terms of the structure is that gastroenterologists tended to use medical records to guide the structure of the consultation (22, 23). However, alternative options have not been elaborated in the literature specific to gastroenterology and we do not know the extent to which specialists in this field come into contact with alternative models used in other contexts of care.

The *voice of the lifeworld* versus the *voice of medicine*

Mishler's concept of *voice* has been used in many health communication research contexts including IBD (by Radley and colleagues). Mishler used the concept to "specify relationships between talk and speakers' underlying frameworks of meaning" (33) and distinguished between the "voice of medicine" and the "voice of the lifeworld". The *voice of medicine*

For peer review only - http://bmjopen.bmj.com/site/about/guidelines.xhtml

represents “the technical-scientific assumptions of medicine” and the *voice of the lifeworld* represents “the natural attitude of everyday life”, according to Mishler (25, p. 13). Radley and colleagues argued that these voices are not mutually exclusive (23). However, the researchers showed that by introducing the *voice of the lifeworld* or what they refer to as the *medico-presentational* regime, the gastroenterologist managed to create a link between IBD as a disease and the patient experience of having IBD and facilitate what can be referred to as shared decision-making.

Nevertheless, the existing IBD communication literature seems to suggest that the dominant voice in the IBD specialist consultations and potentially IBD nurse consultations is the *voice of the medicine*. What strengthens this hypothesis for IBD nurse consultations is the fact that only about half the patients who had searched for medical-related online information prior to the nurse prescription consultation discussed or had the opportunity to discuss that information (20). In IBD specialist consultations, this is construed through the gastroenterologists’ closed-ended questions when assessing patients’ symptoms and disease progression and their interruptions and absence of explicit negotiations of treatment goals during consultations. The dominance of the *voice of the medicine* in IBD specialist consultations has also been demonstrated by the structure of the visits centring around electronic medical records and a checklist of test results, (22). This is despite electronic medical records having the potential to be designed (34) or used (35, 36) in a way that facilitates a patient-centred clinical encounter. What further strengthens the claim that the *voice of the medicine* is generally the dominant voice in IBD specialist consultations is that gastroenterologists in the U.S. asked patients about the impact of IBD on their quality of life or emotional wellbeing in less than a quarter of visits (22). More than half the patients whose quality of life had been impacted by their IBD either did not discuss these impacts with their physician or underplayed the impacts. Furthermore, complete physician-patient alignment in terms of assessment and understanding of the quality of life impacts was present in less than half the cases. In agreement with these findings are the results of the European Federation of Crohn's and Ulcerative Colitis Associations (EFCCA) patient survey in 2007 which showed that less than half of the physicians asked their patients with IBD about their quality of life and less than half of the patients initiated a conversation with their physicians about quality of life (37). Furthermore, research suggested that patients discussed quality of life only if asked directly (38) and were not as comfortable with discussing emotional concerns as their gastroenterologists believed them to be (39), which highlights the value of establishing the *voice of the lifeworld* in conversations with patients with IBD.

Importantly, orienting to the lifeworld in clinical consultation does not mean merely allowing patients to prioritise topics but also means that patients’ evaluations of the relative risks and benefits of different management strategies and the way these fit with their values must also be negotiated. The opportunity to negotiate treatment options in the context of patient values allows patients to potentially reason through their resistance towards the take-up of recommended treatment, which is generally treated as simply a problem (e.g. by Sanders and Linn, as shown in this review) but which other researchers describe as ‘rational action’ on the part of patients (40). An enhanced critical awareness of this literature would benefit IBD research and practice.

From a research design perspective, orienting to the lifeworld could arguably mean establishing research that places the patient at the centre of a network of clinical relationships

For peer review only - http://bmjopen.bmj.com/site/about/guidelines.xhtml

and information sources to do with managing their disease. Sanders and Linn have gone some way towards this by exploring how information outside the clinic is considered in the context of clinical consultation. This approach could be extended to augment our understanding of the roles of nurses, specialists, general practitioners, friends, family, internet information pages, internet support groups and how people with IBD negotiate such networks of information and values.

Goals of treatment, patient knowledge, and the representation of advanced therapies

The existing IBD communication literature demonstrated that treatment goals were not explicitly negotiated by the gastroenterologist with their patient in the consultation. In addition, patients did not seem to be equipped with the necessary knowledge to be able to effectively negotiate the goals of treatment and make a decision. In particular, Rubin and colleagues observed that even though the term *inflammation* was frequently used by the patients and the gastroenterologists in the consultations, patients did not demonstrate clinical understanding of the term and gastroenterologists did not explain its meaning. This is supported by the current general literature of IBD which suggests that patients with IBD do not have as much disease-related knowledge as they need to manage their condition in an ideal way (39, 41-44).

When transparency regarding goals of treatment as well as patients' disease-related knowledge are suboptimal, shared decision-making is compromised. In this context, the way gastroenterologists represent different therapy options in their interactions with patients (intentionally or unintentionally) may be the only available resource for patient decision-making about IBD treatment. However, research has shown that the way clinicians subconsciously represent options can be different from what they think is best (45). This increases the risk of patients choosing an option that disagrees either with their own preferences or the clinical recommendations. A possible avenue for further research is to investigate and compare gastroenterologists' representation of treatment options in the consultation with their opinion about the best treatment option, assessed in a post-visit interview.

The importance IBD-specific health communication research

Earlier in the introduction section, it was argued that it is important to study the details of how clinical communication takes place in the context of IBD and that drawing only on generic principles of health care communication or research involving patients with other health conditions is not adequate for optimising IBD care delivery. The importance of patient engagement, shared decision-making, and patient-centred care in IBD has been emphasised by researchers, clinicians, and institutions for a long time. Different IBD standards recommend the implementation of general principles from health communication research including supporting patients to exercise choice between treatments and follow-up care models, information provision, and education (e.g. Australian and European Standards (46, 47)). Despite that, this review of the very limited literature on observed actual IBD communication suggests that IBD specialist care is not as patient-centred as it could be and shared decision-making does not always occur in the consultations. The review also highlighted an important gap in knowledge, confirming that there is currently a limited

For peer review only - http://bmjopen.bmj.com/site/about/guidelines.xhtml

capacity for evidence-based communication in IBD because there is only rather limited evidence.

Researchers and practitioners can make hypotheses about how results on communication in other diseases transfer to IBD however those hypotheses need to be examined and supported by evidence. In the absence of evidence resulting from the systematic study of clinical communication in IBD, it cannot be certain whether certain recommendations would have the same effect in the specific context of IBD. It is also possible to miss issues that might be discovered in IBD for the first time or those that might be concentrated in IBD but affect other disease groups too. As such, researchers need to team up with the community of IBD practitioners and patients to identify and address the communication issues experienced by them instead of simply doing research on them.

LIMITATIONS

The findings of this systematic review should be interpreted in the light of its limitations. First, the findings could not be integrated to produce cumulative evidence due to the diverse range of included studies in terms of context, theoretical underpinnings, and data types. However, analysis of findings in terms of the linguistic metafunction(s) they address, resulted in the generation of new evidence which revealed the gaps in IBD communication research and provided suggestions for further research as discussed throughout this review. In addition, where possible in the discussion section, links were made between the existing findings to generate new knowledge. A second limitation was that we excluded reports published by abstract only. There were three relevant published abstracts that were excluded from this review. One was related to an included publication (22, 48). One was a 2010 descriptive study of interactions between gastroenterologists and patients with Crohn's disease regarding treatment with biologic therapy (49). Information on any potential eligible publication related to this project was not available at the time of submitting this review. The other excluded published abstract reported the results of an ongoing observational study of 102 IBD clinic consultations conducted by 24 IBD clinicians (10 consultants, 10 IBD nurses and 4 trainees) with the aim to explore variability in the assessment and recording of clinical IBD outcomes during routine practice in England (50). Further work from this project is currently under review at the time of writing this systematic review, according to the first author. The other limitation of this review was that we considered only articles that were in English. However, this limitation did not seem to influence the results of the review. While non-English publications were excluded at the level of database search for PsychINFO and EMBASE, they were included in the database search and later excluded manually by the reviewers for the other five databases. This included 103 papers in 16 languages other than English. None used real-world clinician-patient interaction data in their analysis according to our review of the English translated abstracts. These publications are not reflected in the number of publications reported in this project.

CONCLUSIONS

Further research needs to be conducted to understand the dynamics and details of communication between IBD clinicians (including nurses, consultants and other members of

For peer review only - http://bmjopen.bmj.com/site/about/guidelines.xhtml

the multidisciplinary team) and patients (including adults, adolescents, preconception and pregnant women, etc.) and its effect on patient outcomes. Crucial to such research will be the systematic analysis of clinical interaction with a focus on contributions from all parties, acknowledging that the patient is the centre of a dense network of meanings and relations around their disease, and drawing on a functionally oriented model of language to explore these meanings and relations.

CONTRIBUTORSHIP STATEMENT

NK, ARM and AL conceived the idea of this systematic review project.

NK led the project, developed the protocol, conducted the review, and prepared the first draft of this manuscript with constructive feedback from ARM, AL, SC, RK and AJW on the design of the protocol, the review process, and the manuscript.

RK and AL conducted the title and abstract screening as second reviewers.

ARM provided expert advice and guidance throughout the project and on the manuscript as the senior health communication researcher on this project.

COMPETING INTERESTS

NK, ARM, and AL have received grant support from Janssen.

RK has received research and educational support from Pfizer, Abbvie, Takeda, and Janssen.

AJW has received Honoraria from Takeda, Ferring, Janssen, and Abbvie.

SC is on advisory boards, has received speaker fees, educational support, research support and /or coordinated education meetings for: AbbVie, Celgene, Ferring, Gilead, Janssen, MSD, Orphan/Aspen, Pfizer, and Takeda.

FUNDING

The authors received no specific funding for this work.

DATA SHARING

The data underlying this article will be shared on reasonable request to the corresponding author.

References

1. Mocciaro F, Di Mitri R, Russo G, Leone S, Quercia V. Motivational interviewing in inflammatory bowel disease patients: a useful tool for outpatient counselling. Dig Liver Dis. 2014;46(10):893-7.
2. Mitchell R, Kremer A, Westwood N, Younge L, Ghosh S. Talking about life and IBD: A paradigm for improving patient-physician communication. Journal of Crohn's and Colitis. 2009;3(1):1-3.

For peer review only - http://bmjopen.bmj.com/site/about/guidelines.xhtml

3. Kennedy A, Gask L, Rogers A. Training professionals to engage with and promote self-management. Health Educ Res. 2005;20(5):567-78.
4. Linn AJ, van Weert JCM, van Dijk L, Horne R, Smit EG. The value of nurses' tailored communication when discussing medicines: Exploring the relationship between satisfaction, beliefs and adherence. Journal of Health Psychology. 2016;21(5):798-807.
5. Colombel J-F, D'haens G, Lee W-J, Petersson J, Panaccione R. Outcomes and Strategies to Support a Treat-to-target Approach in Inflammatory Bowel Disease: A Systematic Review. Journal of Crohn's and Colitis. 2019;14(2):254-66.
6. Peyrin-biroulet L, Sandborn W, Sands BE, Reinisch W, Bemelman W, Bryant RV, et al. Selecting Therapeutic Targets in Inflammatory Bowel Disease (STRIDE): Determining Therapeutic Goals for Treat-to-Target. The American Journal of Gastroenterology. 2015;110(9):1324-38.
7. Siegel CA, Lofland JH, Naim A, Gollins J, Walls DM, Rudder LE, et al. Gastroenterologists' Views of Shared Decision Making for Patients with Inflammatory Bowel Disease. Digestive Diseases and Sciences. 2015;60(9):2636-45.
8. Drescher H, Lissoos T, Hajisafari E, Evans ER. Treat-to-Target Approach in Inflammatory Bowel Disease: The Role of Advanced Practice Providers. The Journal for Nurse Practitioners. 2019;15(9):676-81.
9. Moore AR. Language and medicine. In: Thompson G, Bowcher WL, Fontaine L, Schönthal D, editors. The Cambridge Handbook of Systemic Functional Linguistics. Cambridge: Cambridge University Press; 2019. p. 651-88.
10. Karimi N, Moore AR, Lukin A, Kanazaki R, Williams AJ, Connor S. Clinical communication in inflammatory bowel disease: a systematic literature review protocol. BMJ open. 2020;10(11):e039503.
11. Kindell J, Keady J, Sage K, Wilkinson R. Everyday conversation in dementia: a review of the literature to inform research and practice. International journal of language & communication disorders. 2017;52(4):392-406.
12. Parry RH, Land V, Seymour J. Communicating face-to-face about sensitive future matters including end of life: a systematic review of evidence from fine-grained observational research PROSPERO 2011;CRD42011001626.
13. Parry RH, Land V. Systematically reviewing and synthesizing evidence from conversation analytic and related discursive research to inform healthcare communication practice and policy: an illustrated guide. BMC medical research methodology. 2013;13:69.
14. Stortenbeker I, Stommel W, van Dulmen S, Lucassen P, Das E, olde Hartman T. A review on linguistic and interactional aspects in consultations about medically unexplained symptoms. . PROSPERO. 2018;CRD42018095405
15. Halliday MAK, Matthiessen CMIM. Halliday's introduction to functional grammar. London & New York: Routledge; 2014.
16. Matthiessen CMIM. Register in the round: Diversity in a unified theory of register analysis. In: Ghadessy M, editor. Register Analysis: Theory and Practice. London: Pinter; 1993. p. 221–92.
17. Pluye P, Robert E, Cargo M, Bartlett G, O'Cathain A, Griffiths F, et al. Proposal: A mixed methods appraisal tool for systematic mixed studies reviews. Retrieved on [date] from http://mixedmethodsappraisaltoolpublic.pbworks.com. Archived by WebCite® at http://www.webcitation.org/5tTRTc9yJ. 2011.
18. Halliday MAK. Part A. In: Halliday MAK, Hasan R, editors. Language, Context, and Text: Aspects of Language in a Social-Semiotic Perspective. Geelong: Deakin University Press; 1985. p. 3–49.
19. Moher D, Liberati A, Tetzlaff J, Altman DG, The PRISMA Group. Preferred Reporting Items for Systematic Reviews and Meta-Analyses: The PRISMA Statement. PLoS Med. 2009;6(7):e1000097.

For peer review only - http://bmjopen.bmj.com/site/about/guidelines.xhtml

20. Sanders R, Linn AJ. A Mixed Method Study Investigating the Impact of Talking about Patients' Internet Use on Patient-Reported Outcomes. Journal of Health Communication. 2018;23(9):815-23.
21. Linn AJ, van Dijk L, Smit EG, Jansen J, van Weert JC. May you never forget what is worth remembering: the relation between recall of medical information and medication adherence in patients with inflammatory bowel disease. Journal of Crohn's & colitis. 2013;7(11):e543-50.
22. Rubin DT, Dubinsky MC, Martino S, Hewett KA, Panés J. Communication Between Physicians and Patients with Ulcerative Colitis: Reflections and Insights from a Qualitative Study of In-Office Patient-Physician Visits. Inflamm Bowel Dis. 2017;23(4):494-501.
23. Radley A, Mayberry J, Pearce M. Time, space and opportunity in the outpatient consultation: 'the doctor's story'. Soc Sci Med. 2008;66(7):1484-96.
24. Linn AJ, Schouten BC, Sanders R, van Weert JCM, Bylund CL. Talking about Dr. Google: Communication strategies used by nurse practitioners and patients with inflammatory bowel disease in the Netherlands to discuss online health information. Patient Educ Couns. 2020;103(6):1216-22.
25. Leach P, De Silva M, Mountifield R, Edwards S, Chitti L, Fraser RJ, et al. The effect of an inflammatory bowel disease nurse position on service delivery. Journal of Crohn's & colitis. 2014;8(5):370-4.
26. Hunter J, James S, Chan D, Stacey B, Stroud M, Patel P, et al. OC-008 Virtual inflammatory bowel disease clinics in the real world. Gut. 2010;59(Suppl 1):A3-A4.
27. Al-Hillawi L, Blaker P, Harris A, Gurung B, Bedwell T, McEnery C, et al. PTU-075 What is the benefit of telephone and virtual IBD clinics in a DGH? Gut. 2018;67(Suppl 1):A227-A9.
28. Coenen S, Weyts E, Vermeire S, Ferrante M, Noman M, Ballet V, et al. Effects of introduction of an inflammatory bowel disease nurse position on the quality of delivered care. European journal of gastroenterology & hepatology. 2017;29(6):646-50.
29. Hunter J, Claridge A, James S, Chan D, Stacey B, Stroud M, et al. Improving outpatient services: the Southampton IBD virtual clinic. Postgraduate medical journal. 2012;88(1042):487-91.
30. Sechi A, Sawyer E, Ng WSW, Connor SJ. Mo1089 Proving Cost Effectiveness Through Implementation of a Nurse-Led Inflammatory Bowel Disease (IBD) Patient Advice Line and Virtual Clinic (CHEAP). Gastroenterology. 2016;150(4, Supplement 1):S631.
31. Tan SS, Goonawardene N. Internet Health Information Seeking and the Patient-Physician Relationship: A Systematic Review. Journal of medical Internet research. 2017;19(1):e9.
32. Chung JE. Patient-provider discussion of online health information: results from the 2007 Health Information National Trends Survey (HINTS). J Health Commun. 2013;18(6):627-48.
33. Mishler EG. The discourse of medicine : dialectics of medical interviews. Norwood, N.J. [Great Britain]: Ablex Pub. Corp; 1984.
34. Nusbaum NJ. The electronic medical record and Patient-centered care. Online J Public Health Inform. 2011;3(2):ojphi.v3i2.3721.
35. Alkureishi MA, Lee WW, Webb S, Arora V. Integrating Patient-Centered Electronic Health Record Communication Training into Resident Onboarding: Curriculum Development and Post-Implementation Survey Among Housestaff. JMIR Med Educ. 2018;4(1):e1-e.
36. Alkureishi MA, Lee WW, Lyons M, Press VG, Imam S, Nkansah-Amankra A, et al. Impact of Electronic Medical Record Use on the Patient-Doctor Relationship and Communication: A Systematic Review. Journal of general internal medicine. 2016;31(5):548-60.
37. Ghosh S, Mitchell R. Impact of inflammatory bowel disease on quality of life: Results of the European Federation of Crohn's and Ulcerative Colitis Associations (EFCCA) patient survey. Journal of Crohn's & colitis. 2007;1(1):10-20.

For peer review only - http://bmjopen.bmj.com/site/about/guidelines.xhtml

38. Mitchell R, Kremer A, Westwood N, Younge L, Ghosh S. Talking about life and IBD: A paradigm for improving patient–physician communication. Journal of Crohn's and Colitis. 2009;3(1):1-3.
39. Connor SJ, Sechi A, Witcombe D, Fox H. IBD Clinical: Ulcerative Colitis (UC) Narrative Study Australian data: A survey to compare patient and physician perspectives on communication and management of UC. Journal of Gastroenterology and Hepatology. 2018;33:86-115.
40. Donovan JL, Blake DR. Patient non-compliance: deviance or reasoned decision-making? Soc Sci Med. 1992;34(5):507-13.
41. Wardle RA, Mayberry JF. Has patient knowledge of Inflammatory Bowel Disease improved since 1999? Acta Gastroenterol Belg. 2015;78(4):381-5.
42. Siegel CA, Levy CL, MacKenzie TA, Sands BE. Patient perceptions of the risks and benefits of infliximab for the treatment of inflammatory bowel disease. Inflammatory Bowel Diseases. 2008;14(1):1-6.
43. Vegni E, Gilardi D, Bonovas S, Corrò BE, Menichetti J, Leone D, et al. Illness Perception in Inflammatory Bowel Disease Patients is Different Between Patients With Active Disease or in Remission: A Prospective Cohort Study. Journal of Crohn's and Colitis. 2019;13(4):417-23.
44. Wardle RA, Mayberry JF. Patient knowledge in inflammatory bowel disease: the Crohn’s and Colitis Knowledge Score. European Journal of Gastroenterology & Hepatology. 2014;26(1).
45. Lu A, Mohan D, Alexander SC, Mescher C, Barnato AE. The Language of End-of-Life Decision Making: A Simulation Study. J Palliat Med. 2015;18(9):740-6.
46. Crohn’s & Colitis Australia (CCA). Australian IBD Standards: Standards of healthcare for people with inflammatory bowel disease in Australia 2016.
47. Fiorino G, Lytras T, Younge L, Fidalgo C, Coenen S, Chaparro M, et al. Quality of Care Standards in Inflammatory Bowel Diseases: a European Crohn’s and Colitis Organisation [ECCO] Position Paper. Journal of Crohn's and Colitis. 2020;14(8):1037-48.
48. Rubin DT, Dubinsky MC, Martino S, Hewett KA, Panés J, Marren A. Communication Between Physicians and Patients with Ulcerative Colitis: Insights from Studies of Patient-Doctor Visits: 611. Official journal of the American College of Gastroenterology | ACG. 2016;111.
49. Persley K, Eagan C, Waters H, Onofrey M. Gastroenterologist-Patient Communication About Crohn's Disease and Treatment with Biologic Therapy: Results of an In-office Linguistic Study: 1292. Official journal of the American College of Gastroenterology | ACG. 2010;105.
50. Razanskaite V, Williamson P, Young B, Bodger K. P178 Variation in assessment of outcomes for inflammatory bowel disease in routine clinical practice: a mixed-methods study. Journal of Crohn's and Colitis. 2020;14(Supplement_1):S227-S.
51. Jansen J, van Weert J, van der Meulen N, van Dulmen S, Heeren T, Bensing J. Recall in older cancer patients: measuring memory for medical information. Gerontologist. 2008;48(2):149-57.

For peer review only

Table 1. Publication characteristics

Publication	Research question(s)	Condition	Population and eligibility criteria	Country	Design	Measures/analytical framework	Whether part of a bigger study?
(20)	What are the predictors of medication intake behaviour? (implied)	Ulcerative colitis<br><br>Crohn's disease	**Patients** (n=68)<br>• Diagnosed with Crohn's disease or Ulcerative Colitis according to classical clinical, endoscopic, radiographic and/or pathological histological criteria as determined by an experienced gastroenterologist,<br>• Starting Azathioprine, 6-mercaptopurine, Infliximab, Methotrexaat, 6-thioguanine, or Adalimumab, and<br>• Fluent in Dutch<br>**IBD nurses** (n=8)	Netherlands	Mixed method	**Content:** An extensive observation checklist based on comparable studies in oncology<br>**Immediate recall of medical information:** An adapted version of The Netherlands Patient Information Recall Questionnaire (NPIRQ) (51)<br>**Delayed recall of medical information:** An adapted version of the immediate recall questionnaire based on each individual consultation<br>**Percentage of accurate recall:** The ratio of the number of the accurately recalled items to the total number of items discussed coded by two coders using videotaped visits<br>**Total recall score:** The mean recall percentage per patient for immediate and delayed recall<br>**Medication intake behaviour:** A single item self-report measure	Yes - part of a larger study developing an intervention to tailor the communication to IBD patients' needs and barriers to medication intake behaviour
(21)	• How is talking about online information related to patient satisfaction, recall of medical information, and medication adherence?<br>• How many patients seek online health information prior to their consultation? RQ3: How many patients and health-care providers talk about online information and who initiates the discussion?	Ulcerative colitis<br><br>Crohn's disease	**Patients** (n=160)<br>• Diagnosed with Crohn or Ulcerative Colitis<br>• Starting Azathioprine, Methotrexate, Adalimumab, Infliximab, 6-mercaptopurine, or 6-thioguanine<br>• Able to speak and write Dutch<br>**Nurses** (n=8)	Netherlands	Mixed method	**Online Medical Information Seeking Behaviour:** Patients explicitly asked if they used the internet to search for medical information<br>**Patient satisfaction:** A 29-statement scale (4)<br>**Recall of medical information:** A structured telephone interview using an adapted version of the Netherlands Patient Information Recall Questionnaire (NPIRQ) (51)<br>**Medication adherence:** A single item self-report measure<br>**Discussion of online medical information:** Mention of (any combination of) the words *internet*, *Google(d)*, *webpages*, *fora*, *online*, or any other internet-related words during the recorded consultation and the person initiating the discussion	Yes - part of a larger research project developing and testing a theoretical and evidence- based tailored multimedia intervention to improve medication adherence in patients with IBD
(22)	How are the opportunities for the integration of patients' concerns enabled or hindered by the gastroenterologist? (implied)	Ulcerative colitis<br><br>Crohn's disease	Main study<br>**Physicians** (n=3), **Patients** (n=16)<br>Reported case study<br>• A woman patient (with her young daughter) with a registrar<br>• The same patient with a consultant<br>• A woman in her mid- 30s,	UK	Qualitative	**Patient expectations of the future consultation and patient reflection after the consultation:** Episodic interviews<br>**Interaction:** the notion of *proto-story*	Yes – part of a project investigating how and when the concerns of IBD patients are included within the treatment protocols of their doctors

			with a long history of ulcerative colitis who had become a mother for the first time six months previously (with her baby) with the consultant				
(23)	• Is there alignment between patients and physicians in terms of the impact of disease on the patient's QoL? (implied)<br>• What is perceived to be treatment success in patients' and doctors' view? (implied)<br>• In which visits the physician asked patients about QoL?<br>• Which patients were experiencing QoL and emotional impacts due to their disease and, of these, which patients discussed these impacts during the visit?<br>• Which patients demonstrated evidence of resigning themselves to active symptoms as acceptable (i.e., 'a new normal' or 'learned helplessness')?<br>• In which visits the physician interrupted the patient and the number of interruptions?<br>• In which visits the physician asked primarily closed-ended questions to assess symptoms?<br>• In which visits the physician conveyed a significant gap in their perception of conventional therapies for UC (e.g., 5-ASA and corticosteroids) and advanced therapies (e.g., immunomodulators	Ulcerative colitis	US study<br>**Gastroenterologists** (n=15)<br>• board-certified gastroenterologists, and spending at least 75% of their professional time in direct patient care<br>• be in full-time practice for 3 to 30 years,<br>• primarily see patients in an office or private practice setting<br>• see at least 25 patients diagnosed with UC in a typical month<br>• have initiated biologic therapy for at least 15% of their patients with UC<br>**Patients** (n=40)<br>• 18 years old or older<br>• Diagnosed with moderate-to-severe ulcerative colitis for ≥1 year<br>• At least one flare within the past year according to participating gastroenterologists<br>• Had previously received or were currently receiving therapy with 5-ASA and/or corticosteroids<br>• Fluent in English<br>• No cognitive impairment<br>EU study<br>**Gastroenterologists** (n= 8)<br>• Spending at least 60% of their time in direct patient care<br>• Primarily treating adults<br>• Main speciality being gastroenterology	US and Europe (Italy, Germany, France)	Mixed method	**Patient and physician attitudes toward and framing of immunomodulatory and biologic therapies:**<br>EU: Qualitative analysis of visits and post-visit interviews<br>US:<br>• Number of visits in which the physician conveyed a significant gap between conventional therapies and advanced therapies<br>• Number of visits in which the physician did not compare or discuss advance therapies<br>• Position of advanced therapies in the sequence of treatment in the visits in which they were discussed<br>• Number of visits in which gastroenterologists framed biologic therapy as a last resort<br>• Number of visits in which it was clear that gastroenterologists had previously discussed advanced options<br>• Number of patients who were not currently or previously prescribed advanced therapies and who discussed advanced therapy options during the post-visit interview and their appraisal of advanced therapies during the interview<br>**Patients and physicians' use and understanding of the term *inflammation***:<br>• Number of visits in which the term *inflammation* (or a variation thereof) was used by the physician<br>• Context in which the term *inflammation* was used by the physicians<br>• Number of visits in which the physician described what they meant by the term and explained the relevance of the term<br>• Number of visits in which the patient used the term *inflammation* (or variations)<br>• Number of interviews in which the patient used the term *inflammation* and context in which the patients used the term *inflammation*<br>**Patient and physician description of treatment success:**<br>Analysed in terms of absence of clinical symptoms	No

	and biologic therapies)?<br>• In which visits advanced therapies were framed as a last resort and thus considered only if absolutely necessary?<br>• In which visits advanced therapies were discussed and where they were positioned in the treatment sequence?<br>• Which patients considered advanced therapies as more appropriate for more severe stages of disease than they believed they were experiencing?		• Planning to discuss immunosuppressant or biologic treatment with their patient at their next visit<br>• In full-time practice for 3 to 30 years<br>• Primarily seeing patients in an office or private practice setting<br>• Seeing at least 25 patients diagnosed with ulcerative colitis in a typical month<br>• Have initiated biologic therapy for at least 15% of their patients with ulcerative colitis<br>• **Patients** (n=28)<br>18 years old or older<br>• Diagnosed with moderate-to-severe ulcerative colitis for ≥1 year<br>• At least one flare within the past year according to participating gastroenterologists<br>• Had previously received or were currently receiving therapy with 5-ASA and/or corticosteroids<br>• No cognitive impairment			(clinical remission) or absence of inflammation (endoscopic remission)<br>**Further measures in the US study:**<br>• **Alignment between patients and physicians on QoL impacts:** (1) complete alignment; (2) partial alignment; (3) no alignment<br>• **Number of visits in which the physician asked patients about QoL or emotional impact of the disease**<br>• **Number of patients who were experiencing QoL and emotional impacts due to their disease and number of patients who did not discuss or underplay these impacts during the visit**<br>• **Number of patients who demonstrated evidence of resigning themselves to active symptoms as acceptable (i.e., 'a new normal' or 'learned helplessness')**<br>• **Percentage of words spoken by the participants and the length of each visit**<br>• **Number of visits in which the physician interrupted the patient and the number of interruptions**<br>• **Number of visits in which the physician asked primarily closed-ended questions to assess symptoms**	

1. Jansen J, van Weert J, van der Meulen N, van Dulmen S, Heeren T, Bensing J. Recall in older cancer patients: measuring memory for medical information. Gerontologist. 2008;48(2):149-57.
2. Linn AJ, van Weert JCM, van Dijk L, Horne R, Smit EG. The value of nurses' tailored communication when discussing medicines: Exploring the relationship between satisfaction, beliefs and adherence. Journal of Health Psychology. 2016;21(5):798-807.

Table 2. Characteristics of the interaction data

Paper	Year	Country	Setting	Number of sites	Corpus size	Average length of consultations	Whether series or one off	Participants	Patient demographics
(20)	2013	Netherlands	Nurse-patient prescribing consultation - Hospital	6	59 video-recorded and 9 audio-recorded consultations (68 consultations)	29.5 min (SD = 8.5)	One off	Nurses (n=8)<br>Patients (n=68)	Gender (N): Female: 42, Male: 26<br>Age M (SD): 40.5 (14.9)<br>Condition*: CD: 54, UC: 13, Unknown:1<br>Diagnosed in years M (SD): 9.6 (10.3)<br>Education level: Low: 18, Moderate: 24, High: 26<br>Living arrangements: Alone: 16, With partner: 16, With partner and child(ren) 16, With Child(ren): 8, Other: 12<br>Employed: yes: 51<br>Ethnicity: Dutch: 62
(21)	2018	Netherlands	Nurse-patient prescribing consultation - Hospital	6	165 video-recorded consultations (5 excluded from analysis due to lack of demographic data): 160 consultations	Not stated	One off	Nurses (n=8)<br>Patients (n=160)	Gender (N): Female: 92, Male: 68<br>Age M (SD): 43.10 (15.33)<br>Condition (N): CD: 101, UC: 49, other: 10<br>Diagnosed in years M (SD): 11.61 (10.55), Range: 1.5-47.1<br>Educational level: Low: 36, Moderate: 61, High: 63
(22)	2008	UK	Outpatient clinic - Hospital	1	3x16 video-recorded consultations (assumed info, not explicitly stated in the article)	Not stated	Series (3 consultations from each patient)	Physicians (n=3)<br>Patients (n=16)	Not stated
(23)	2017	US and Europe (Italy, Germany, France)	outpatient clinics - community-based practices	Not stated	US study: 40 visits<br>EU study: 28 visits	15 min (U.S. research)	One off	US study.:<br>Gastroenterologists (n=15)<br>Patients (n=40)<br>EU study:<br>Gastroenterologists (n=8, Italy: 5, Germany: 2, France: 1)<br>Patients (n=28)	US study:<br>Gender: Female: 21, Male: 19<br>Age M (Range): 49 (20-83)<br>EU study:<br>Gender: Female: 11, Male: 17<br>Age:<br>18-34 years old: 4 patients<br>35-54 years old: 14 patients<br>55-74 years old: 4 patients<br>Unavailable: 6 patients

* CD: Crohn's disease, UC: ulcerative colitis

For peer review only

Figure Legends

Figure 1. The screening and inclusion process

Figure 2. Findings of articles investigating nurse-patient communication categorised by linguistic metafunction

Figure 3. Findings of articles investigating gastroenterologist-patient communication categorised by linguistic metafunction

For peer review only

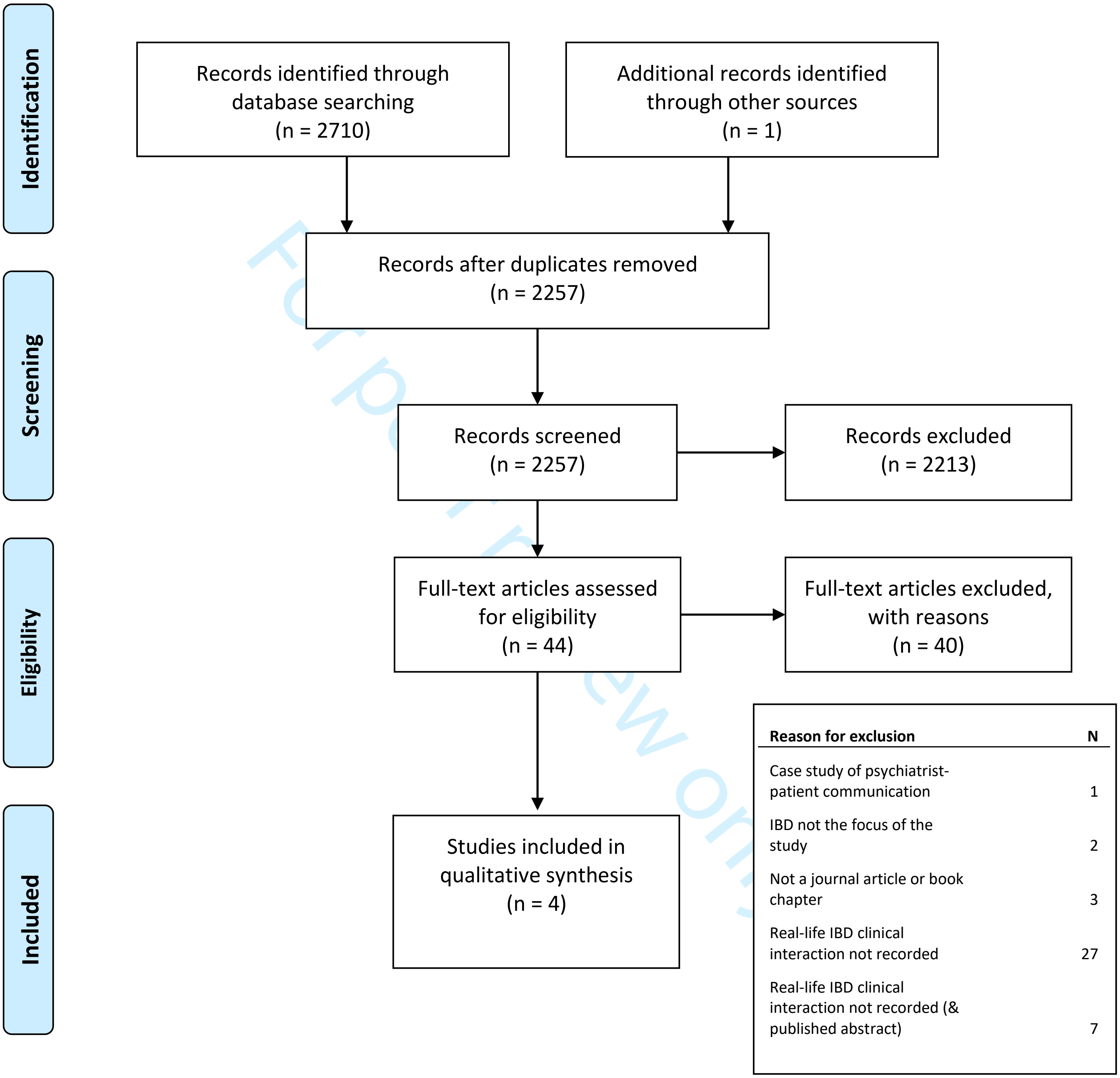

Figure 1. The screening and inclusion process

Interpersonal   Ideational   Textual

Nurse-patient clinical communication

Sanders, R. and A. J. Linn (2018)

Direct reference to online health information: In 46.8% of the 95 consultations (n=44) , online health information was directly referenced. Overall, reference to online health information was almost evenly initiated by patients (n=33; 55%) and health-care providers (n=27; 45%).

Patients who directly refered to online health information during consultation were more satisfied with the consultation (mean=0.86; SD=.61) compared to patients who searched online but did not talk about it (mean=1.30; SD=1.13, Mdiff=-0.43, p=.041, d=0.48).

There was no significant relation between reference to online health information and recall of medical information (Mdiff=0.12, p=0.310).

There was no significant direct relation between patient satisfaction and the recall of medical information (Mdiff=-0.13, p=0.270)
For patients referring to online health information, higher satisfaction with the consultation related to higher recall of medical information, compared to not discussing it (Mdiff=0.32, p=0.054).

Linn, A. J., et al. (2013)

Medical information discussed (% of consultations in which each category was discussed): Purpose of the medication (89.7%); Name of medication (98.5%); Duration of treatment (48.5%); Frequency of administration (97.1%); Influence of the medication on the immune system (88.2%); When to expect an effect from the medication (80.9%); The need for blood monitoring (80.9%); Side effects (100%); When to contact the nurse (94.1%); The possibility of experiencing side effects (47.1%); The impact of the medication on the patients' daily life (79.4%); medication intake behaviour advice (44.1%)

Medical information recalled (%): Purpose of the medication (32.7%); Name of medication (83.6%); Duration of treatment (59.8%); Frequency administration (85.9%); Influence of the medication on the immune system (69.2%); When to expect an effect from the medication (66.8%); The need for blood monitoring (63.6%); Side effects (26.7%); When to contact the nurse (39.2%); The possibility of experiencing side effects (78.8%); The impact of the medication on the patients' daily life (5.5%); Medication intake behaviour advice (15%).

Delayed recall of medical information was significantly related to self-reported medication intake behavior (β=0.37, p=0.007). Patients with lower recall scores three weeks after the consultation rated themselves as less adherent than the patients with higher recall scores.

For peer review only - http://bmjopen.bmj.com/site/about/guidelines.xhtml

Figure 2. Findings of articles investigating nurse-patient communication categorised by linguistic metafunction

Interpersonal | Ideational | Textual

Gastroenterologist-patient clinical communication

Radley, A., et al. (2008)

The way doctors sit, make references to their notes, ask questions, provide information, construct the concept of time and space, their posture, tone of voice, speed of talking, pauses, and interruptions mediate their view of the pateint's illness: medico-scientific view versus medicopresentational view.

Effect on patient experience: In the consultation in which the doctor held a medico-scientific view of the illness, the patient noted that the doctor “did not really listen to what she had to say”.
In the consultation in which the doctor had a medicopresentational view of the illness, the patient noted that the doctor put her “on the right road really”.

Rubin, D. T., et al. (2017)

Question asking: Gastroenterologists typically used closed questions (73% of the visits, n=29) when assessing patients’ symptoms and disease progression. Completion of electronic medical records often guided the dialogue and thus eye contact and elaboration of symptoms by the patient were limited.

Discussing QoL and emotional impact: Gastroenterologists asked patients about their QoL or emotional well-being in 23% (n=9) of visits. Fifty-five percent of the 31 patients who were experiencing QoL impacts either did not discuss these impacts during their visit (n=10; 32%) or underplayed the impacts (n=7; 23%). Of patients experiencing symptoms, most demonstrated evidence of resigning themselves to a 'new normal'.

Inflammation **:** The term *inflammation* (or a variation thereof) was used by gastroenterologists in 60% (n=24) of visits and an average of 4 times per visit. Gastroenterologists described what they meant by the term in only one (2.5%) of the visits and explained the relevance of the term in 3 (7.5%). Patients used the term *inflammation* (or variations) in 13% (n=5) of visits. During post-visit interviews, patients voluntarily used the term in 48% (n=19) of cases, but none described what they meant by the term and none demonstrated clinical understanding of it. Patients’ use of the term was most frequently to justify treatment (30% of all visits; n=12) or in the assessment of their disease (15%; n=6).

Appraisal of advanced therapies: US gastroenterologists conveyed a significant risk level gap between conventional therapies and advanced therapies in 58% of visits (n=23). In 53% of visits (n=21), gastroenterologists framed biologic therapy as a last resort, to be considered only if absolutely necessary. The position of immunomodulatory in the treatment sequence was always undifferentiated.
EU gastroenterologist generally framed immunomodulatory therapies as an intermediate therapy for patients who had failed treatment with conventional therapies. Biologic therapy was positioned at the end of the treatment sequence, to be considered once other options had been considered and/or exhausted. Physicians positioned biologic agents as potent, effective therapies with known risks.

Goals of care: During visits with patients for whom the gastroenterologist viewed treatment success as endoscopic remission (10 visits), the gastroenterologist clearly communicated this goal in 40% of cases (n=4).

Attitudes toward treatment success: Gastroenterologists described treatment success for most patients as clinical remission (73% of cases; n=29). In 25% of cases (n=10), gastroenterologists described treatment success as endoscopic remission; in half of these 10 cases, endoscopic remission was an additional goal and in the other half, endoscopic remission was the only goal. Most patients (78%; n=31) described treatment success as an absence of clinical symptoms, i.e., clinical remission.

Interruptions: Gastroenterologists interrupted patients at least once in 63% (n=25) of visits. The average number of interruptions per visit was 1.5.

Contribution: Gastroenterologists spoke, on average, 61% of the words spoken during the visit and patients contributed 36% of the words spoken during visits.

Advanced therapies: Biologic therapies were not discussed in 40% of visits (n=16). Immunomodulatory therapy was discussed in 40% of visits (n=16). In 60% of visits (n=24), it was clear that gastroenterologists had previously discussed advanced options.

Patient attitude toward advanced therapies: Of the patients who were not currently or previously prescribed advanced therapies and who discussed advanced therapy options during the post-visit interview, all (n=17) considered advanced therapies as more appropriate for stages of disease more severe than their own.

EU gastroenterologists often structured the visit around a checklist of test results .
Fr US gastroenterologist, completion of electronic medical records often guided the dialogue and thus eye contact and elaboration of symptoms by the patient were limited.

The term *inflammation* was most frequently (n=20) used during the assessment of the patient’s disease. Gastroenterologists also used the term during treatment justification (n=9) and disease education (n=8), among other contexts.

Physician-patient alignment (in terms of the asssessment of the QoL impacts): Complete alignment was demonstrated for 40% (n=16) of the consultations, partial alignment for 43% (n=17) of the consultation, and no alignment for 18% (n=7) of the consultations. Of the 17 consultations in which partial alignment was demonstrated, 12 were due to the gastroenterologist underestimating the QoL impacts; 3 were due to gastroenterologist overestimation; and 2 were due to the gastroenterologist and patient mentioning different QoL impacts.

Effect of physician interruption on the length of the visit: The average visit length increased with the number of interruptions.

For peer review only - http://bmjopen.bmj.com/site/about/guidelines.xhtml

Figure 3. Findings of articles investigating gastroenterologist-patient communication categorised by linguistic metafunction

Supplementary Table 1. Data items included in the data extraction template

- **Participant characteristics**
  - Participants and numbers
  - Participant socio-demographics
  - Additional health status information (e.g. pregnancy status, adult versus adolescent)
- **Disease characteristics**
  - Type of IBD
  - Disease activity
  - Disease phenotype
  - Extraintestinal manifestations
- **Study characteristics**
  - Year of research
  - Country of research
  - Research setting and type of consultation
  - Stated aims
  - Aims relevant to the review
  - Study design
  - Outcomes and measures
  - Stated findings
  - Stated conclusions
- **Consultation data and analysis characteristics**
  - Corpus size and number of consultations/episodes analysed in the study
  - Average length of consultations
  - One-off consultation or series
  - Data type (audio or video)
  - Method of linguistic data analysis (sociolinguistics, conversation analysis, content analysis, etc.)
  - Linguistic component/s analysed

For peer review only

Supplementary Table 2a. Analyses of risk of bias (tailored framework)

	Credibility									Reliability	
**Article**	**Type of consultation data**	**Size of consultation data analysed**	**Sample size justification**	**Linguistic component(s) analysed**	**Linguistic data analysis tool(s)**	**Demographic data**	**Additional contextual information**		**Arguments/findings supported by textual data (1-3: 1=NO and 3 =YES)**	**Unit of analysis**	**Second coder**
(20)	Nurse-patient consultation	59 video-recorded, 9 audio-recorded consultations (68 consultations)	No	Topic	An observation checklist (presence/absence)	Yes	No		1	No	Yes
(21)	Nurse-patient consultation	95 video-recorded consultations	No	Lexis	Presence/absence of a list of words and the speaker	Yes	No		1	No	No
(22)	Gastroenterologist-patient consultation	2 video-recorded consultation	No	Sitting, Reference to notes, Representation of space and time	*Proto-story*	No	Yes	• Patient interviews<br>• Information on patients' personal life and consultant level of experience	2	No	No
(23)	Gastroenterologist-patient consultation	68 audio- and video-recorded consultations	No	Lexis, Topic, Question asking, Contribution to conversation, Interruptions, framing	Lexis: presence/absence, context and co-text, speaker<br>Topic: presence/absence, speaker<br>Question-asking: open-ended vs. closed<br>Contribution to conversation: Percentage of words spoken by the participants and the length of each visit<br>Interruptions: Number<br>Framing: Position in treatment sequence	Yes	Yes	• Patient and consultant interviews	2	No	No

For peer review only

Supplementary Table 2b. Analysis of risk of bias (Mixed Methods Appraisal Tool)

	Mixed Methods Appraisal Tool (MMAT) – Version 2011	Sanders, R. and A. J. Linn (2018)	Linn, A. J., et al. (2013)
Quantitative nonrandomized	Are participants (organizations) recruited in a way that minimizes selection bias?	No - 63.12% of participants had CD, 77.5% of the participants were moderately or highly educated	No - 62.3% of participants were female, 79.4% had CD, 73.5% were moderately or highly educated, 91.2% were Dutch
	Are measurements appropriate (clear origin, or validity known, or standard instrument; and absence of contamination between groups when appropriate) regarding the exposure/intervention and outcomes?	Online Medical Information Seeking Behaviour: Patients explicitly asked if they used the internet to search for medical information ("yes" or "no") - **Yes**<br>Patient satisfaction: a 29-statement scale (Linn, van Weert, van Dijk, Horne, & Smit, 2016) – **Can't tell, tool could not be accessed**<br>Recall of medical information: a structured telephone interview using an adapted version of the Netherlands Patient Information Recall Questionnaire (NPIRQ) (Jansen et al., 2008) - **Yes**<br>Medication adherence: A single item self-report measure (Linn et al., 2013) - **Yes**	Immediate recall of medical information: An adapted version of The Netherlands Patient Information Recall Questionnaire (NPIRQ) - **Yes**<br>Delayed recall of medical information: An adapted version of the immediate recall questionnaire based on each individual consultation - **Yes (immediate and delayed recall measures were not the same)**<br>Percentage of accurate recall: The ratio of the number of the accurately recalled items to the total number of items discussed coded by two coders using videotaped visits - **Yes**<br>Total recall score: the mean recall percentage per patient for immediate and delayed recall - **Yes**<br>Medication intake behaviour: A single item self-report measure ("Please indicate on a scale from 1 to 10 the extent to which you are taking the medication as prescribed)" - **Yes**
	In the groups being compared (exposed vs. non-exposed; with intervention vs. without; cases vs. controls), are the participants comparable, or do researchers take into account (control for) the difference between these groups?	Can't tell	Yes
	Are there complete outcome data (80% or above), and, when applicable, an acceptable response rate (60% or above), or an acceptable follow-up rate for cohort studies (depending on the duration of follow-up)?	Yes	Yes
Qualitative	Are the sources of qualitative data (archives, documents, informants, observations) relevant to address the research question (objective)?	Yes	Yes
	Is the process for analyzing qualitative data relevant to address the research question (objective)?	Yes	Yes
	Is appropriate consideration given to how findings relate to the context, e.g., the setting, in which the data were collected?	No - Discussion of online health information was regarded as a dichotomy. How the online information was discussed was not studied.	No - the effect of contextual factors was not considered in analysing the content of the consultations.
	Is appropriate consideration given to how findings relate to researchers' influence, e.g., through their interactions with participants?	Yes	The likely influence of video-recording consultation on participants' behaviour was not addressed.
Mixed methods	Is the mixed methods research design relevant to address the qualitative and quantitative research questions (or objectives), or the qualitative and	Yes	Yes

For peer review only

	quantitative aspects of the mixed methods question (or objective)?		
	Is the integration of qualitative and quantitative data (or results*) relevant to address the research question (objective)?	Yes	Yes
	Is appropriate consideration given to the limitations associated with this integration, e.g., the divergence of qualitative and quantitative data (or results*) in a triangulation design?	N/A	N/A
Screening questions	Are there clear qualitative and quantitative research questions (or objectives*), or a clear mixed methods question (or objective*)?	Yes	Yes
	Do the collected data allow address the research question (objective)? E.g., consider whether the follow-up period is long enough for the outcome to occur (for longitudinal studies or study components).	Yes	Yes

For peer review only

PRISMA 2009 Checklist

Section/topic	#	Checklist item	Reported on page #
**TITLE**			
Title	1	Identify the report as a systematic review, meta-analysis, or both.	1
**ABSTRACT**			
Structured summary	2	Provide a structured summary including, as applicable: background; objectives; data sources; study eligibility criteria, participants, and interventions; study appraisal and synthesis methods; results; limitations; conclusions and implications of key findings; systematic review registration number.	2
**INTRODUCTION**			
Rationale	3	Describe the rationale for the review in the context of what is already known.	3
Objectives	4	Provide an explicit statement of questions being addressed with reference to participants, interventions, comparisons, outcomes, and study design (PICOS).	3
**METHODS**			
Protocol and registration	5	Indicate if a review protocol exists, if and where it can be accessed (e.g., Web address), and, if available, provide registration information including registration number.	3
Eligibility criteria	6	Specify study characteristics (e.g., PICOS, length of follow-up) and report characteristics (e.g., years considered, language, publication status) used as criteria for eligibility, giving rationale.	3-4
Information sources	7	Describe all information sources (e.g., databases with dates of coverage, contact with study authors to identify additional studies) in the search and date last searched.	4
Search	8	Present full electronic search strategy for at least one database, including any limits used, such that it could be repeated.	4
Study selection	9	State the process for selecting studies (i.e., screening, eligibility, included in systematic review, and, if applicable, included in the meta-analysis).	4
Data collection process	10	Describe method of data extraction from reports (e.g., piloted forms, independently, in duplicate) and any processes for obtaining and confirming data from investigators.	4
Data items	11	List and define all variables for which data were sought (e.g., PICOS, funding sources) and any assumptions and simplifications made.	4
Risk of bias in individual studies	12	Describe methods used for assessing risk of bias of individual studies (including specification of whether this was done at the study or outcome level), and how this information is to be used in any data synthesis.	4
Summary measures	13	State the principal summary measures (e.g., risk ratio, difference in means).	N/A
Synthesis of results	14	Describe the methods of handling data and combining results of studies, if done, including measures of consistency (e.g., I^2) for each meta-analysis.	4

For peer review only

PRISMA 2009 Checklist

Section/topic	#	Checklist item	Reported on page #
Risk of bias across studies	15	Specify any assessment of risk of bias that may affect the cumulative evidence (e.g., publication bias, selective reporting within studies).	N/A
Additional analyses	16	Describe methods of additional analyses (e.g., sensitivity or subgroup analyses, meta-regression), if done, indicating which were pre-specified.	4
**RESULTS**			
Study selection	17	Give numbers of studies screened, assessed for eligibility, and included in the review, with reasons for exclusions at each stage, ideally with a flow diagram.	5
Study characteristics	18	For each study, present characteristics for which data were extracted (e.g., study size, PICOS, follow-up period) and provide the citations.	5
Risk of bias within studies	19	Present data on risk of bias of each study and, if available, any outcome level assessment (see item 12).	5
Results of individual studies	20	For all outcomes considered (benefits or harms), present, for each study: (a) simple summary data for each intervention group (b) effect estimates and confidence intervals, ideally with a forest plot.	6 and figures 2 & 3
Synthesis of results	21	Present results of each meta-analysis done, including confidence intervals and measures of consistency.	N/A
Risk of bias across studies	22	Present results of any assessment of risk of bias across studies (see Item 15).	N/A
Additional analysis	23	Give results of additional analyses, if done (e.g., sensitivity or subgroup analyses, meta-regression [see Item 16]).	6 and figures 2 & 3
**DISCUSSION**			
Summary of evidence	24	Summarize the main findings including the strength of evidence for each main outcome; consider their relevance to key groups (e.g., healthcare providers, users, and policy makers).	6-10
Limitations	25	Discuss limitations at study and outcome level (e.g., risk of bias), and at review-level (e.g., incomplete retrieval of identified research, reporting bias).	10
Conclusions	26	Provide a general interpretation of the results in the context of other evidence, and implications for future research.	10
**FUNDING**			
Funding	27	Describe sources of funding for the systematic review and other support (e.g., supply of data); role of funders for the systematic review.	1

From: Moher D, Liberati A, Tetzlaff J, Altman DG, The PRISMA Group (2009). Preferred Reporting Items for Systematic Reviews and Meta-Analyses: The PRISMA Statement. PLoS Med 6(7): e1000097. doi:10.1371/journal.pmed1000097

For more information, visit: **www.prisma-statement.org**.

For peer review only - http://bmjopen.bmj.com/site/about/guidelines.xhtml

BMJ Open

Clinical communication in inflammatory bowel disease: a systematic review of the study of clinician-patient dialogue to inform research and practice

Journal:	*BMJ Open*
Manuscript ID	bmjopen-2021-051053.R1
Article Type:	Original research
Date Submitted by the Author:	13-Jun-2021
Complete List of Authors:	Karimi, Neda; Ingham Institute; University of New South Wales, South Western Sydney Clinical School<br>Kanazaki, Ria; Liverpool Hospital, Gastroenterology Department; University of New South Wales, Ingham Institute of Applied Medical Research<br>Lukin, Annabelle; Macquarie University, Department of Linguistics, Faculty of Medicine, Health and Human Sciences, Macquarie University<br>Moore, Alison; University of Wollongong Faculty of Law Humanities and the Arts<br>Williams, Astrid-Jane; Liverpool Hospital, Department of Gastroenterology; Ingham Institute,<br>Connor, Susan; University of New South Wales, South Western Sydney Clinical School; Liverpool Hospital, Department of Gastroenterology
**Primary Subject Heading**:	Gastroenterology and hepatology
Secondary Subject Heading:	Communication, Medical education and training, Patient-centred medicine, Qualitative research, Sociology
Keywords:	Inflammatory bowel disease < GASTROENTEROLOGY, QUALITATIVE RESEARCH, SOCIAL MEDICINE

SCHOLARONE™
Manuscripts

I, the Submitting Author has the right to grant and does grant on behalf of all authors of the Work (as defined in the below author licence), an exclusive licence and/or a non-exclusive licence for contributions from authors who are: i) UK Crown employees; ii) where BMJ has agreed a CC-BY licence shall apply, and/or iii) in accordance with the terms applicable for US Federal Government officers or employees acting as part of their official duties; on a worldwide, perpetual, irrevocable, royalty-free basis to BMJ Publishing Group Ltd ("BMJ") its licensees and where the relevant Journal is co-owned by BMJ to the co-owners of the Journal, to publish the Work in this journal and any other BMJ products and to exploit all rights, as set out in our licence.

The Submitting Author accepts and understands that any supply made under these terms is made by BMJ to the Submitting Author unless you are acting as an employee on behalf of your employer or a postgraduate student of an affiliated institution which is paying any applicable article publishing charge ("APC") for Open Access articles. Where the Submitting Author wishes to make the Work available on an Open Access basis (and intends to pay the relevant APC), the terms of reuse of such Open Access shall be governed by a Creative Commons licence – details of these licences and which Creative Commons licence will apply to this Work are set out in our licence referred to above.

Other than as permitted in any relevant BMJ Author's Self Archiving Policies, I confirm this Work has not been accepted for publication elsewhere, is not being considered for publication elsewhere and does not duplicate material already published. I confirm all authors consent to publication of this Work and authorise the granting of this licence.

For peer review only

Clinical communication in inflammatory bowel disease: a systematic review of the study of clinician-patient dialogue to inform research and practice

Neda Karimi[1,3], PhD; Ria Kanazaki[1,2,3], MD; Annabelle Lukin[4], PhD; Alison Rotha Moore[5], PhD; Astrid-Jane Williams[1,2], MD; Susan J Connor[1,2,3], PhD

[1]Ingham Institute for Applied Medical Research, Sydney, Australia
[2]Department of Gastroenterology, Liverpool Hospital, Sydney, Australia
[3]South Western Sydney Clinical School, The University of NSW, Sydney, Australia
[4]Department of Linguistics, Faculty of Medicine, Health and Human Sciences, Macquarie University, Sydney, Australia
[5]School of Humanities and Social Inquiry, Faculty of Law, Humanities and the Arts, The university of Wollongong, Wollongong, Australia

**Corresponding author:**	Neda Karimi, PhD
**Postal address:**	Gastroenterology Research Group, Ingham Institute, Liverpool Hospital, Locked Bag 7103, LIVERPOOL BC NSW 1871 Australia
**Email Address:**	neda.karimi@unsw.edu.au
**Phone:**	+61 (02) 8738 9247

For peer review only - http://bmjopen.bmj.com/site/about/guidelines.xhtml

ABSTRACT

Objectives: This systematic review aims to investigate what is currently known about the characteristics of interactions between patients with inflammatory bowel disease (IBD) and their clinicians and its effect on patient outcomes.

Data sources: Scopus, PubMed, Embase, Communication Abstracts, Health & Society, Linguistics and Language Behavior Abstracts, and PsycINFO were systematically searched from inception to June 2021.

Study eligibility criteria: Peer-reviewed journal articles and book chapters in English investigating the characteristics of naturally occurring interactions between clinicians that manage IBD patients and patients with IBD during recorded consultations were included.

Study appraisal and synthesis methods: Risk of bias was assessed using a specifically developed quality assessment tool, grounded in linguistic theory, and the Mixed Methods Appraisal Tool. A narrative synthesis guided by the linguistic-theoretical concept of metafunction was performed to synthesise the findings.

Results: Of the 2883 abstracts reviewed five formed the basis of the review. Interactions between IBD nurses and patients have been mostly characterised in terms of information provision regarding prescribed medications without consideration of the interpersonal aspect. Discussing online medical information with nurses has been shown to improve patient satisfaction. Analyses of gastroenterologist-patient interactions have concentrated on the clinical relationship which has been shown to be disease-centred. Shared decision-making in ulcerative colitis has been shown to be compromised due to lack of transparency regarding treatment goals.

Limitations: This review did not include articles in languages other than English. Cumulative evidence could not be produced due to the small number of included studies and diversity of contexts, theories, and data types.

Conclusions and implications of key findings: There is a paucity of systematic research on naturally occurring clinical communication in IBD and its effect on outcomes**.** Further research needs to be done to address this knowledge gap.

Systematic review registration number: PROSPERO registration number CRD42020169657

STRENGTHS AND LIMITATIONS OF THIS STUDY

- This systematic review summarises and interprets the available evidence on clinical communication in IBD by assessing evidence resulting from investigations of authentic clinical interactions in IBD (real-life data) rather than investigations of self-reported data (perceptions and attitudes).
- The review consults a diverse range of databases, including databases with special focus on medicine, health, psychology, communication and linguistics, to identify eligible studies.
- The synthesis of the results is guided by a well-established theory of language.
- The review does not include articles in languages other than English.

For peer review only

For peer review only - http://bmjopen.bmj.com/site/about/guidelines.xhtml

- The review does not provide a complete overview of clinical communication in IBD and its effect due to limited evidence in this space.

INTRODUCTION

The main space in which clinicians and patients manage care, negotiate roles, and make decisions is their interaction during consultations. Understanding communication between clinicians and patients in this space and its existing variations is crucial for understanding the bigger picture of how disease is managed.

In inflammatory bowel disease (IBD), clinical communication is argued to affect patient satisfaction, treatment adherence, patient quality of life, disease management, and self-management (1-4). In addition, in tandem with the recognition of the importance of minimising disease activity at an early stage in IBD - using a 'treat-to-target' (T2T) management approach (5, 6), research promoting shared decision-making in IBD has gained momentum (7). A T2T approach involves personalised care and early interventions aimed at delaying or preventing disease progression, preventing bowel damage, and promoting mucosal healing (6). It is a collaborative approach involving the physician (and the multidisciplinary team) and the patient (6). It involves joint risk-benefit assessment and decision-making, monitoring, and optimising therapy to achieve disease control and symptom improvement (5, 8). As a result, discourses around communication and the role of the patient as a key stakeholder in decision-making have gained recognition in IBD research (8).

Currently, studies that investigate communication between IBD clinicians and patients through investigation of the "clinician-patient interface" (9) - i.e. projects that investigate interactions between patients and clinicians, rather than patients' perceptions of clinical communication - are less common. The current review ascertains the existing knowledge in this area in order to inform the field, identify areas that require further investigation, and position this literature within current IBD care practice and research. The main objective is to identify, organise and summarise systematically what is currently known about (a) the characteristics of conversations between clinicians that manage IBD and patients with IBD, and (b) how clinical discussion affects health outcomes in IBD. A secondary objective is to identify the different approaches to the analysis of clinical interaction in IBD, the methodological trends, and the gaps for future research. Underpinning both of these objectives, it is also our aim to show the importance of studying the details of how clinical communication takes place in the specific context of IBD and why drawing only on highly generic principles of health care communication or research involving patients with other health conditions is not sufficient for maximising the delivery of quality care in IBD.

METHOD

Study selection, data extraction, quality assessment, and synthesis

The protocol for this systematic review is registered with PROSPERO (CRD42020169657) and separately published (10).

Published peer-reviewed journal articles and book chapters in English that investigated the characteristics of naturally occurring interactions between clinicians that manage IBD patients and patients with IBD during recorded consultations were included in this review.

For peer review only - http://bmjopen.bmj.com/site/about/guidelines.xhtml

Self-report studies that explored clinicians' and patients' attitudes, or beliefs about clinical interaction only were excluded. Target participant groups included patients with IBD and clinicians that manage IBD patients in primary and secondary health care (e.g. general practitioners, IBD specialists, IBD nurses), complementary and alternative medicine (e.g. acupuncturists, traditional Chinese medicine practitioners), or allied health (e.g. dietitians). Health care providers whose primary treatment includes the interaction itself (e.g. psychotherapists) were not included. Studies in which eligible participant groups were present but IBD was not the focus of the study were also excluded.

Scopus, PubMed, Embase, Communication Abstracts, Health & Society, Linguistics and Language Behavior Abstracts (LLBA), and PsycINFO were searched from inception to June 2021. The reference lists of eligible as well as excluded but relevant publications were screened. Subject matter experts were consulted to ensure inclusiveness. The search strategy is available in our published protocol (10).

Titles and abstracts were screened by three reviewers (NK, AL, and RK). The reviewers identified eligible publications based on their title and abstract, compared their lists of selected publications, and resolved any discrepancies prior to the full-text review. Full-text publications were screened for final inclusion by the reviewers with complete agreement between the reviewers.

Data were extracted from included articles by one reviewer (NK) and checked by the review team for accuracy. The template used for data extraction was developed in consultation with the existing health communication and linguistics literature including previous systematic literature reviews of this kind (9, 11-14) and included the data items presented in Supplementary Table 1.

Risk of bias was assessed using a template designed by the review team in consultation with previous systematic reviews of this kind (13) and compatible with the principles of systemic functional linguistics (15, 16) which offers a conceptual framework for the architecture of language, tools to measure the different components of language, and methodological approaches for principled selection of data and data analysis tools to reduce the description bias and increase credibility. For more information on the assessment of risk of bias refer to the protocol paper (10). In addition to the tailored template, to assess risk of bias in mixed-methods studies in which the analysis of talk was a secondary aim, the Mixed Methods Appraisal Tool (MMAT) (17) was used.

The extracted data were summarised and compared in overview tables and figures and are interpreted in a narrative synthesis. Halliday's concept of metafunction was used to classify the communicative aspects analysed by the included studies. The concept of metafunction is derived from the view that language has evolved in and through the social contexts in which it functions, and this contextual pressure in its evolution produced three main organising principles for language. All languages, despite their considerable variation, give their speakers resources for (1) construing human experiences (ideational metafunction), (2) enacting personal and social relationships (interpersonal metafunction), and (3) organising a discursive flow and creating cohesion and continuity (textual metafunction) (18). Each linguistic feature analysed by the included papers was coded depending on which of these functions it served. Classifying the included publications based on the metafunction

For peer review only - http://bmjopen.bmj.com/site/about/guidelines.xhtml

addressed has the advantage of identifying systematically the aspects of IBD clinical communication which have been described as well as those that are not yet explored.

Ethical statement

Ethics reviews and approval were not required for this systematic review as no primary data were collected.

Patient and public involvement

Patients or the public were not involved in the design and conduct of this systematic review.

RESULTS

Study selection and study characteristics

Of the 3482 search results identified as potentially relevant to our research question, 2883 unique publications were reviewed, and five papers from four individual studies fulfilled the inclusion criteria. Figure 1 illustrates the screening and inclusion process.

Insert Figure 1. Here (19)

Included studies were conducted in six countries (The Netherlands, UK, Italy, Germany, France, and USA) and in two institutional contexts (outpatient IBD nurse consultation, outpatient specialist consultation). Studies were published between 2008 and 2020. The characteristics of included studies are described in Table 1 and the characteristics of the interaction data used in these studies are summarised in Table 2.

Risk of bias within studies

Following assessment of risk of bias (Supplementary Tables 2a and 2b), all five publications were included in data synthesis.

Two publications (20, 21) were mixed-methods correlational studies mainly examining selected predictive variables and their relationship with medication adherence. The aim in these projects was to identify the predictors of medication intake behaviour which was facilitated through investigation of nurse-patient interaction. A related third publication was a mixed-methods study that investigated the communicative strategies employed by patients and IBD nursed to discuss online health information (22). The fourth and fifth publications that were included in this review (23, 24) were descriptive studies and detailed in terms of their linguistic analysis. Both studies combined analysis of recorded IBD specialist consultations with patient interviews. Rubin and colleagues (23) conducted physician interviews, in addition to patient interviews, and investigated physician-patient alignment.

The analysis of the interactions in the two nurse-focused publications aiming to investigate predictors of medication intake behaviour was limited to determining the presence or absence of preselected pieces of medicines information. The analysis of clinical talk by Sanders and Linn (20) was limited to coding the interaction in terms of the presence of the words *internet*, *Google(d)*, *webpages*, *fora*, *online*, or any other internet-related words and the person initiating such discussion. Similarly, the analysis in the 2013 Linn et al. study (21) included coding the data in terms of the presence or absence of certain pieces of information regarding prescribed medication. Other than the demographic data, no contextual information was collected in the two publications. Nor were the analyses accompanied by examples of

For peer review only - http://bmjopen.bmj.com/site/about/guidelines.xhtml

interactions. However, since the collected data and the processes for analysing qualitative data were relevant to address the research questions (refer to MMAT analysis), these two publications were included in the review. The analysis of talk in in the third IBD nurse communication study included in this review was more detailed than in the two earlier studies. This third study used grounded theory and quantitative content analysis to code the communicative data. Qualitative findings were quantified and accompanied by examples of single conversational turns or excerpts from single turns that instantiated them.

The studies that were focused on IBD specialist consultations provided examples from their textual data to support the findings, however this was confined to single conversational turns or excerpts from single turns in Rubin et al. (23). Radley and colleagues (24) included extra contextual data such as the consultant's level of experience and information on the personal life of the patient in their analysis, and included evidence from excerpts of gastroenterologist-patient interactions that illustrate patterns such as turn-taking and how topics are initiated and responded. However, these excerpts were taken from three consultations only.

None of the five studies provided sample size justification or mentioned use of a unit of analysis in their analysis. Only Linn et al. 2013 and Linn et al. 2020 determined interobserver reliability for text coding.

Results: Nurse-patient communication

The majority of the nurse-patient communication studies in this review (20, 21) focused on the effect of communicating information regarding prescribed medicines (ideational metafunction) on patient medication intake behaviour. Only one recent study in our dataset looked at the dynamics of the nurse-patient relationship (interpersonal metafunction) and no study has looked at the structure of nurse-led consultations or the continuity of dialogue and how topics introduced by patients are maintained or ignored (textual metafunction).

Figure 2 summarises the findings of those publications in terms of the characteristics of nurse-patient interactions and their effects on patient outcomes and categorises these findings in terms of the linguistic function they address.

Insert Figure 2 Here

Results: Gastroenterologist-patient communication

Studies that investigated gastroenterologist-patient communication were different from those that investigated nurse-patient communication in their focus and objectives. The studies mainly focused on the interpersonal relationship between the gastroenterologist and the patient and the treatment decision-making process with the aim to reduce the distance between the patient and the consultant.

Figure 3 summarises the findings of the two articles that described the characteristics of the gastroenterologist-patient communication and categorises these findings in terms of the linguistic function they investigate.

Insert Figure 3 Here

For peer review only

DISCUSSION

Nurses as transmitters of information

Historically, the IBD health communication literature identified nurses only as transmitters of information about new medications, assuming their interactions with patients to have only an ideational function. Using this methodology, data relating to turn-taking, boundaries (who initiates a topic and who closes a topic), evaluation and attitudes, elaboration, or engagement are not captured. The interpersonal function of the nurse-patient communication in IBD has more recently started to attract the attention of IBD researchers. The 2020 Linn et al. study looked at the way online health information-seeking is discussed by patients and nurse practitioners during prescription consultations and investigated their interactions from an interpersonal perspective (22).

Nurses are at the forefront of IBD healthcare, providing medical and psycho-social care (including but not limited to provision of information) via IBD advicelines and virtual clinics and are at times the mediator or gatekeeper between the patient and the gastroenterologist (25-30). Understanding the dynamics of the IBD nurse-patient interaction as a bidirectional process that both responds to the context at hand and reshapes it will help develop effective communication strategies and identify communicative risks. In achieving this understanding, a robust account of variation in nurse-patient interaction is crucial since small changes in ways of speaking can produce big differences in how a context is experienced and interpreted by patients. The current literature, seemed to use 'common sense' rather than evidence, to provide nurse-patient communication recommendations. For example, Linn et al.'s (21) suggestion that signposting and categorising information can improve recall was not grounded in the findings of their study. These recommendations seem to be hypotheses that would need testing.

Discussion of online health information

Sanders and Linn showed that only about half the patients who had searched for medical-related online information prior to the nurse prescription consultation discussed or had the opportunity to discuss that information (20). The 2020 Linn et al. paper revealed that patients and nurses were equally likely to initiate the discussion of online information-seeking; and that they did so often by using a general strategy such as making a general statement on the part of the patient and exploring whether (and what) the patient searched online before the consultation on the part of the nurse (22). Nurses, however, varied in terms of broaching the discussion of online health information with some initiating more frequently than others, according to this study. Barriers to discussing online health information are known from studies outside IBD to include clinician's resistance towards discussing such information, patient concerns surrounding disapproval by the clinician or fear of embarrassment (31). It has also been shown outside IBD that men are more likely than women to have a conversation regarding online information with physicians (32). Internet health information seeking and discussing such information during the consultation can empower patients and give them a sense of control and confidence when talking to their clinician (31). It can also help them gain greater clarity, orientation, and certainty regarding their own health and lead to higher patient satisfaction as shown in this review, so it seems important to increase opportunities for such discussion. Research has shown that the clinician's communication skills and their open response to a less hierarchised relationship as a result of a patient

For peer review only - http://bmjopen.bmj.com/site/about/guidelines.xhtml

discussing online health information is important in forming a positive relationship with the patient (31).

Gastroenterologist-patient communication

Gastroenterologist-patient communication has been conceptualised around the interpersonal relationship between the gastroenterologist and the patient and its role in the decision-making process. However, the focus of the literature is on the gastroenterologist's contribution to the dialogue with less emphasis on contributions from the patient. Furthermore, little information is provided on the structure of the IBD specialist consultations. What is known, in terms of the structure is that gastroenterologists tended to use medical records to guide the structure of the consultation (23, 24). However, alternative options have not been elaborated in the literature specific to gastroenterology and we do not know the extent to which specialists in this field come into contact with alternative models used in other contexts of care.

The *voice of the lifeworld* versus the *voice of medicine*

Mishler's concept of *voice* has been used in many health communication research contexts including IBD (by Radley and colleagues). Mishler used the concept to "specify relationships between talk and speakers' underlying frameworks of meaning" (33) and distinguished between the "voice of medicine" and the "voice of the lifeworld". The *voice of medicine* represents "the technical-scientific assumptions of medicine" and the *voice of the lifeworld* represents "the natural attitude of everyday life", according to Mishler (33, p. 13). Radley and colleagues argued that these voices are not mutually exclusive (24). However, the researchers showed that by introducing the *voice of the lifeworld* or what they refer to as the *medico-presentational* regime, the gastroenterologist managed to create a link between IBD as a disease and the patient experience of having IBD and facilitate what can be referred to as shared decision-making.

Nevertheless, the existing IBD communication literature seems to suggest that the dominant voice in the IBD specialist consultations and potentially IBD nurse consultations is the *voice of the medicine*. What strengthens this hypothesis for IBD nurse consultations is the fact that only about half the patients who had searched for medical-related online information prior to the nurse prescription consultation discussed or had the opportunity to discuss that information (20). In IBD specialist consultations, this is construed through the gastroenterologists' closed-ended questions when assessing patients' symptoms and disease progression and their interruptions and absence of explicit negotiations of treatment goals during consultations. The dominance of the *voice of the medicine* in IBD specialist consultations has also been demonstrated by the structure of the visits centring around electronic medical records and a checklist of test results, (23). This is despite electronic medical records having the potential to be designed (34) or used (35, 36) in a way that facilitates a patient-centred clinical encounter. What further strengthens the claim that the *voice of the medicine* is generally the dominant voice in IBD specialist consultations is that gastroenterologists in the U.S. asked patients about the impact of IBD on their quality of life or emotional wellbeing in less than a quarter of visits (23). More than half the patients whose quality of life had been impacted by their IBD either did not discuss these impacts with their physician or underplayed the impacts. Furthermore, complete physician-patient alignment in terms of assessment and understanding of the quality of life impacts was present in less than half the cases. In agreement with these findings are the results of the European Federation of

For peer review only - http://bmjopen.bmj.com/site/about/guidelines.xhtml

Crohn's and Ulcerative Colitis Associations (EFCCA) patient survey in 2007 which showed that less than half of the physicians asked their patients with IBD about their quality of life and less than half of the patients initiated a conversation with their physicians about quality of life (37). Furthermore, research suggested that patients discussed quality of life only if asked directly (38) and were not as comfortable with discussing emotional concerns as their gastroenterologists believed them to be (39), which highlights the value of establishing the *voice of the lifeworld* in conversations with patients with IBD.

Importantly, orienting to the lifeworld in clinical consultation does not mean merely allowing patients to prioritise topics but also means that patients' evaluations of the relative risks and benefits of different management strategies and the way these fit with their values must also be negotiated. The opportunity to negotiate treatment options in the context of patient values allows patients to potentially reason through their resistance towards the take-up of recommended treatment, which is generally treated as simply a problem (e.g. by Sanders and Linn, as shown in this review) but which other researchers describe as 'rational action' on the part of patients (40). An enhanced critical awareness of this literature would benefit IBD research and practice.

From a research design perspective, orienting to the lifeworld could arguably mean establishing research that places the patient at the centre of a network of clinical relationships and information sources to do with managing their disease. Sanders and Linn have gone some way towards this by exploring how information outside the clinic is considered in the context of clinical consultation. This approach could be extended to augment our understanding of the roles of nurses, specialists, general practitioners, friends, family, internet information pages, internet support groups and how people with IBD negotiate such networks of information and values.

Goals of treatment, patient knowledge, and the representation of advanced therapies

The existing IBD communication literature demonstrated that treatment goals were not explicitly negotiated by the gastroenterologist with their patient in the consultation. In addition, patients did not seem to be equipped with the necessary knowledge to be able to effectively negotiate the goals of treatment and make a decision. In particular, Rubin and colleagues observed that even though the term *inflammation* was frequently used by the patients and the gastroenterologists in the consultations, patients did not demonstrate clinical understanding of the term and gastroenterologists did not explain its meaning. This is supported by the current general literature of IBD which suggests that patients with IBD do not have as much disease-related knowledge as they need to manage their condition in an ideal way (39, 41-44).

When transparency regarding goals of treatment as well as patients' disease-related knowledge are suboptimal, shared decision-making is compromised. In this context, the way gastroenterologists represent different therapy options in their interactions with patients (intentionally or unintentionally) may be the only available resource for patient decision-making about IBD treatment. However, research has shown that the way clinicians subconsciously represent options can be different from what they think is best (45). This increases the risk of patients choosing an option that disagrees either with their own

For peer review only - http://bmjopen.bmj.com/site/about/guidelines.xhtml

preferences or the clinical recommendations. A possible avenue for further research is to investigate and compare gastroenterologists' representation of treatment options in the consultation with their opinion about the best treatment option, assessed in a post-visit interview.

The importance IBD-specific health communication research

Earlier in this paper, we argued that it is important to study the details of how clinical communication takes place in the context of IBD and that drawing only on generic principles of health care communication or research involving patients with other health conditions is not adequate for optimising IBD care delivery. The importance of patient engagement, shared decision-making, and patient-centred care in IBD has been emphasised by researchers, clinicians, and institutions for a long time. Different IBD standards recommend the implementation of general principles from health communication research including supporting patients to exercise choice between treatments and follow-up care models, information provision, and education (e.g. Australian and European Standards (46, 47)). Despite that, this review of the very limited literature on observed actual IBD communication suggests that IBD specialist care is not as patient-centred as it could be and shared decision-making does not always occur in the consultations. The review also highlighted an important gap in knowledge, confirming that there is currently a limited capacity for evidence-based communication in IBD because there is only rather limited evidence.

Researchers and practitioners can make hypotheses about how results on communication in other diseases transfer to IBD however those hypotheses need to be examined and supported by evidence. In the absence of evidence resulting from the systematic study of clinical communication in IBD, it cannot be certain whether certain recommendations would have the same effect in the specific context of IBD. It is also possible to miss issues that might be discovered in IBD for the first time or those that might be concentrated in IBD but affect other disease groups too. As such, researchers need to team up with the community of IBD practitioners and patients to identify and address the communication issues experienced by them instead of simply doing research on them.

Summary of findings

This systematic review reveals that there has been insufficient attention to naturally occurring consultation dialogue in the research on IBD. The review findings suggest that historically IBD nurses have been identified in the IBD literature solely as transmitters of information about new medications. However, this has started to change recently. There is a new strand of research in the IBD literature that acknowledges and investigates the interpersonal meanings exchanged in IBD nurse consultations. Findings so far suggest the importance of nurses eliciting discussion about what kind of online information patients use to help them understand IBD medications and to situate illness and treatment within their lives. Turning to physician-patient communication, our review suggests that IBD care remains unhelpfully disease-centred because of a tightly structured consultation around the consultant's agenda including the completion of electronic medical records and the review of test results and because the patient's quality of life is not fully addressed during the consultation. In addition, shared decision-making in IBD specialist consultations seems to be compromised due to the lack of explicit negotiation around goals of therapy. Information on the different structural components of IBD nurse or specialist consultations is not available in the literature.

For peer review only - http://bmjopen.bmj.com/site/about/guidelines.xhtml

LIMITATIONS

The findings of this systematic review, however, should be interpreted in the light of its limitations. First, the findings could not be integrated to produce cumulative evidence due to the diverse range of included studies in terms of context, theoretical underpinnings, and data types. However, analysis of findings in terms of the linguistic metafunction(s) they address, resulted in the generation of new evidence which revealed the gaps in IBD communication research and provided suggestions for further research as discussed throughout this review. In addition, where possible in the discussion section, links were made between the existing findings to generate new knowledge. A second limitation was that we excluded reports published by abstract only. There were three relevant published abstracts that were excluded from this review. One was related to an included publication (23, 48). One was a 2010 descriptive study of interactions between gastroenterologists and patients with Crohn's disease regarding treatment with biologic therapy (49). Information on any potential eligible publication related to this project was not available at the time of submitting this review. The other excluded published abstract reported the results of an ongoing observational study of 102 IBD clinic consultations conducted by 24 IBD clinicians (10 consultants, 10 IBD nurses and 4 trainees) with the aim to explore variability in the assessment and recording of clinical IBD outcomes during routine practice in England (50). Further work from this project is currently under review at the time of writing this systematic review, according to the first author. The other limitation of this review was that we considered only articles that were in English. However, this limitation did not seem to influence the results of the review. While non-English publications were excluded at the level of database search for PsychINFO and EMBASE, they were included in the database search and later excluded manually by the reviewers for the other five databases. This included 103 papers in 16 languages other than English. None used real-world clinician-patient interaction data in their analysis according to our review of the English translated abstracts. These publications are not reflected in the number of publications reported in this project.

CONCLUSIONS

Further research needs to be conducted to understand the dynamics and details of communication between IBD clinicians (including nurses, consultants and other members of the multidisciplinary team) and patients (including adults, adolescents, preconception and pregnant women, etc.) and its effect on patient outcomes. Crucial to such research will be the systematic analysis of clinical interaction with a focus on contributions from all parties including the patient, the consultant, IBD nurses, the dietitian, and even family or friends attending with the patient, rather than profiling only what clinicians do. This kind of approach allows interactions between the patient and the gastroenterologist, for example, to be interpreted in the context of the patient's interactions with other members of the multidisciplinary team. Such research acknowledges that the patient is the centre of a dense network of meanings and relations around their disease and draws on a functionally oriented model of language to explore these meanings and relations.

For peer review only - http://bmjopen.bmj.com/site/about/guidelines.xhtml

CONTRIBUTORSHIP STATEMENT

NK, ARM and AL conceived the idea of this systematic review project.

NK led the project, developed the protocol, conducted the review, and prepared the first draft of this manuscript with constructive feedback from ARM, AL, SC, RK and AJW on the design of the protocol, the review process, and the manuscript.

RK and AL conducted the title and abstract screening as second reviewers.

ARM provided expert advice and guidance throughout the project and on the manuscript as the senior health communication researcher on this project.

COMPETING INTERESTS

NK, ARM, and AL have received grant support from Janssen.

RK has received research and educational support from Pfizer, Abbvie, Takeda, and Janssen.

AJW has received Honoraria from Takeda, Ferring, Janssen, and Abbvie.

SC is on advisory boards, has received speaker fees, educational support, research support and /or coordinated education meetings for: AbbVie, Celgene, Ferring, Gilead, Janssen, MSD, Orphan/Aspen, Pfizer, and Takeda.

FUNDING

The authors received no specific funding for this work.

DATA SHARING

The data underlying this article will be shared on reasonable request to the corresponding author.

References

1. Mocciaro F, Di Mitri R, Russo G, Leone S, Quercia V. Motivational interviewing in inflammatory bowel disease patients: a useful tool for outpatient counselling. Dig Liver Dis. 2014;46(10):893-7.
2. Mitchell R, Kremer A, Westwood N, Younge L, Ghosh S. Talking about life and IBD: A paradigm for improving patient-physician communication. Journal of Crohn's and Colitis. 2009;3(1):1-3.
3. Kennedy A, Gask L, Rogers A. Training professionals to engage with and promote self-management. Health Educ Res. 2005;20(5):567-78.
4. Linn AJ, van Weert JCM, van Dijk L, Horne R, Smit EG. The value of nurses' tailored communication when discussing medicines: Exploring the relationship between satisfaction, beliefs and adherence. Journal of Health Psychology. 2016;21(5):798-807.
5. Colombel J-F, D'haens G, Lee W-J, Petersson J, Panaccione R. Outcomes and Strategies to Support a Treat-to-target Approach in Inflammatory Bowel Disease: A Systematic Review. Journal of Crohn's and Colitis. 2019;14(2):254-66.

For peer review only

6. Peyrin-biroulet L, Sandborn W, Sands BE, Reinisch W, Bemelman W, Bryant RV, et al. Selecting Therapeutic Targets in Inflammatory Bowel Disease (STRIDE): Determining Therapeutic Goals for Treat-to-Target. The American Journal of Gastroenterology. 2015;110(9):1324-38.
7. Siegel CA, Lofland JH, Naim A, Gollins J, Walls DM, Rudder LE, et al. Gastroenterologists' Views of Shared Decision Making for Patients with Inflammatory Bowel Disease. Digestive Diseases and Sciences. 2015;60(9):2636-45.
8. Drescher H, Lissoos T, Hajisafari E, Evans ER. Treat-to-Target Approach in Inflammatory Bowel Disease: The Role of Advanced Practice Providers. The Journal for Nurse Practitioners. 2019;15(9):676-81.
9. Moore AR. Language and medicine. In: Thompson G, Bowcher WL, Fontaine L, Schönthal D, editors. The Cambridge Handbook of Systemic Functional Linguistics. Cambridge: Cambridge University Press; 2019. p. 651-88.
10. Karimi N, Moore AR, Lukin A, Kanazaki R, Williams AJ, Connor S. Clinical communication in inflammatory bowel disease: a systematic literature review protocol. BMJ Open. 2020;10(11):e039503.
11. Kindell J, Keady J, Sage K, Wilkinson R. Everyday conversation in dementia: a review of the literature to inform research and practice. International journal of language & communication disorders. 2017;52(4):392-406.
12. Parry RH, Land V, Seymour J. Communicating face-to-face about sensitive future matters including end of life: a systematic review of evidence from fine-grained observational research PROSPERO 2011;CRD42011001626.
13. Parry RH, Land V. Systematically reviewing and synthesizing evidence from conversation analytic and related discursive research to inform healthcare communication practice and policy: an illustrated guide. BMC medical research methodology. 2013;13:69.
14. Stortenbeker I, Stommel W, van Dulmen S, Lucassen P, Das E, olde Hartman T. A review on linguistic and interactional aspects in consultations about medically unexplained symptoms. . PROSPERO. 2018;CRD42018095405
15. Halliday MAK, Matthiessen CMIM. Halliday's introduction to functional grammar. London & New York: Routledge; 2014.
16. Matthiessen CMIM. Register in the round: Diversity in a unified theory of register analysis. In: Ghadessy M, editor. Register Analysis: Theory and Practice. London: Pinter; 1993. p. 221–92.
17. Pluye P, Robert E, Cargo M, Bartlett G, O'Cathain A, Griffiths F, et al. Proposal: A mixed methods appraisal tool for systematic mixed studies reviews. Retrieved on [date] from http://mixedmethodsappraisaltoolpublic.pbworks.com. Archived by WebCite® at http://www.webcitation.org/5tTRTc9yJ. 2011.
18. Halliday MAK. Part A. In: Halliday MAK, Hasan R, editors. Language, Context, and Text: Aspects of Language in a Social-Semiotic Perspective. Geelong: Deakin University Press; 1985. p. 3–49.
19. Moher D, Liberati A, Tetzlaff J, Altman DG, The PRISMA Group. Preferred Reporting Items for Systematic Reviews and Meta-Analyses: The PRISMA Statement. PLoS Med. 2009;6(7):e1000097.
20. Sanders R, Linn AJ. A Mixed Method Study Investigating the Impact of Talking about Patients' Internet Use on Patient-Reported Outcomes. Journal of Health Communication. 2018;23(9):815-23.
21. Linn AJ, van Dijk L, Smit EG, Jansen J, van Weert JC. May you never forget what is worth remembering: the relation between recall of medical information and medication adherence in patients with inflammatory bowel disease. Journal of Crohn's & colitis. 2013;7(11):e543-50.
22. Linn AJ, Schouten BC, Sanders R, van Weert JCM, Bylund CL. Talking about Dr. Google: Communication strategies used by nurse practitioners and patients with inflammatory bowel disease in the Netherlands to discuss online health information. Patient education and counseling. 2020;103(6):1216-22.

For peer review only

23. Rubin DT, Dubinsky MC, Martino S, Hewett KA, Panés J. Communication Between Physicians and Patients with Ulcerative Colitis: Reflections and Insights from a Qualitative Study of In-Office Patient-Physician Visits. Inflamm Bowel Dis. 2017;23(4):494-501.
24. Radley A, Mayberry J, Pearce M. Time, space and opportunity in the outpatient consultation: 'the doctor's story'. Soc Sci Med. 2008;66(7):1484-96.
25. Leach P, De Silva M, Mountifield R, Edwards S, Chitti L, Fraser RJ, et al. The effect of an inflammatory bowel disease nurse position on service delivery. Journal of Crohn's & colitis. 2014;8(5):370-4.
26. Hunter J, James S, Chan D, Stacey B, Stroud M, Patel P, et al. OC-008 Virtual inflammatory bowel disease clinics in the real world. Gut. 2010;59(Suppl 1):A3-A4.
27. Al-Hillawi L, Blaker P, Harris A, Gurung B, Bedwell T, McEnery C, et al. PTU-075 What is the benefit of telephone and virtual IBD clinics in a DGH? Gut. 2018;67(Suppl 1):A227-A9.
28. Coenen S, Weyts E, Vermeire S, Ferrante M, Noman M, Ballet V, et al. Effects of introduction of an inflammatory bowel disease nurse position on the quality of delivered care. European journal of gastroenterology & hepatology. 2017;29(6):646-50.
29. Hunter J, Claridge A, James S, Chan D, Stacey B, Stroud M, et al. Improving outpatient services: the Southampton IBD virtual clinic. Postgraduate medical journal. 2012;88(1042):487-91.
30. Sechi A, Sawyer E, Ng WSW, Connor SJ. Mo1089 Proving Cost Effectiveness Through Implementation of a Nurse-Led Inflammatory Bowel Disease (IBD) Patient Advice Line and Virtual Clinic (CHEAP). Gastroenterology. 2016;150(4, Supplement 1):S631.
31. Tan SS, Goonawardene N. Internet Health Information Seeking and the Patient-Physician Relationship: A Systematic Review. Journal of medical Internet research. 2017;19(1):e9.
32. Chung JE. Patient-provider discussion of online health information: results from the 2007 Health Information National Trends Survey (HINTS). J Health Commun. 2013;18(6):627-48.
33. Mishler EG. The discourse of medicine : dialectics of medical interviews. Norwood, N.J. [Great Britain]: Ablex Pub. Corp; 1984.
34. Nusbaum NJ. The electronic medical record and Patient-centered care. Online J Public Health Inform. 2011;3(2):ojphi.v3i2.3721.
35. Alkureishi MA, Lee WW, Webb S, Arora V. Integrating Patient-Centered Electronic Health Record Communication Training into Resident Onboarding: Curriculum Development and Post-Implementation Survey Among Housestaff. JMIR Med Educ. 2018;4(1):e1-e.
36. Alkureishi MA, Lee WW, Lyons M, Press VG, Imam S, Nkansah-Amankra A, et al. Impact of Electronic Medical Record Use on the Patient-Doctor Relationship and Communication: A Systematic Review. Journal of general internal medicine. 2016;31(5):548-60.
37. Ghosh S, Mitchell R. Impact of inflammatory bowel disease on quality of life: Results of the European Federation of Crohn's and Ulcerative Colitis Associations (EFCCA) patient survey. Journal of Crohn's & colitis. 2007;1(1):10-20.
38. Mitchell R, Kremer A, Westwood N, Younge L, Ghosh S. Talking about life and IBD: A paradigm for improving patient–physician communication. Journal of Crohn's and Colitis. 2009;3(1):1-3.
39. Connor SJ, Sechi A, Witcombe D, Fox H. IBD Clinical: Ulcerative Colitis (UC) Narrative Study Australian data: A survey to compare patient and physician perspectives on communication and management of UC. Journal of Gastroenterology and Hepatology. 2018;33:86-115.
40. Donovan JL, Blake DR. Patient non-compliance: deviance or reasoned decision-making? Soc Sci Med. 1992;34(5):507-13.
41. Wardle RA, Mayberry JF. Has patient knowledge of Inflammatory Bowel Disease improved since 1999? Acta Gastroenterol Belg. 2015;78(4):381-5.
42. Siegel CA, Levy CL, MacKenzie TA, Sands BE. Patient perceptions of the risks and benefits of infliximab for the treatment of inflammatory bowel disease. Inflammatory Bowel Diseases. 2008;14(1):1-6.

For peer review only

43. Vegni E, Gilardi D, Bonovas S, Corrò BE, Menichetti J, Leone D, et al. Illness Perception in Inflammatory Bowel Disease Patients is Different Between Patients With Active Disease or in Remission: A Prospective Cohort Study. Journal of Crohn's and Colitis. 2019;13(4):417-23.
44. Wardle RA, Mayberry JF. Patient knowledge in inflammatory bowel disease: the Crohn's and Colitis Knowledge Score. European Journal of Gastroenterology & Hepatology. 2014;26(1).
45. Lu A, Mohan D, Alexander SC, Mescher C, Barnato AE. The Language of End-of-Life Decision Making: A Simulation Study. J Palliat Med. 2015;18(9):740-6.
46. Crohn's & Colitis Australia (CCA). Australian IBD Standards: Standards of healthcare for people with inflammatory bowel disease in Australia 2016.
47. Fiorino G, Lytras T, Younge L, Fidalgo C, Coenen S, Chaparro M, et al. Quality of Care Standards in Inflammatory Bowel Diseases: a European Crohn's and Colitis Organisation [ECCO] Position Paper. Journal of Crohn's and Colitis. 2020;14(8):1037-48.
48. Rubin DT, Dubinsky MC, Martino S, Hewett KA, Panés J, Marren A. Communication Between Physicians and Patients with Ulcerative Colitis: Insights from Studies of Patient-Doctor Visits: 611. Official journal of the American College of Gastroenterology | ACG. 2016;111.
49. Persley K, Eagan C, Waters H, Onofrey M. Gastroenterologist-Patient Communication About Crohn's Disease and Treatment with Biologic Therapy: Results of an In-office Linguistic Study: 1292. Official journal of the American College of Gastroenterology | ACG. 2010;105.
50. Razanskaite V, Williamson P, Young B, Bodger K. P178 Variation in assessment of outcomes for inflammatory bowel disease in routine clinical practice: a mixed-methods study. Journal of Crohn's and Colitis. 2020;14(Supplement_1):S227-S.

For peer review only

Table 1. Publication characteristics

Publication	Research question(s)	Condition	Population and eligibility criteria	Country	Design	Measures/analytical framework	Whether part of a bigger study?
(20)	What are the predictors of medication intake behaviour? (implied)	Ulcerative colitis<br><br>Crohn's disease	**Patients** (n=68)<br>• Diagnosed with Crohn's disease or ulcerative colitis according to classical clinical, endoscopic, radiographic and/or pathological histological criteria as determined by an experienced gastroenterologist,<br>• Starting Azathioprine, 6-mercaptopurine, Infliximab, Methotrexaat, 6-thioguanine, or Adalimumab, and<br>• Fluent in Dutch<br>**IBD nurses** (n=8)	Netherlands	Mixed methods	**Content:** An extensive observation checklist based on comparable studies in oncology<br>**Immediate recall of medical information:** An adapted version of The Netherlands Patient Information Recall Questionnaire (NPIRQ)[1]<br>**Delayed recall of medical information:** An adapted version of the immediate recall questionnaire based on each individual consultation<br>**Percentage of accurate recall:** The ratio of the number of the accurately recalled items to the total number of items discussed coded by two coders using videotaped visits<br>**Total recall score:** The mean recall percentage per patient for immediate and delayed recall<br>**Medication intake behaviour:** A single item self-report measure	Yes - part of a larger study developing an intervention to tailor the communication to IBD patients' needs and barriers to medication intake behaviour
(21)	• How is talking about online information related to patient satisfaction, recall of medical information, and medication adherence?<br>• How many patients seek online health information prior to their consultation? RQ3: How many patients and health-care providers talk about online information and who initiates the discussion?	Ulcerative colitis<br><br>Crohn's disease	**Patients** (n=160)<br>• Diagnosed with Crohn or Ulcerative Colitis<br>• Starting Azathioprine, Methotrexate, Adalimumab, Infliximab, 6-mercaptopurine, or 6-thioguanine<br>• Able to speak and write Dutch<br>**Nurses** (n=8)	Netherlands	Mixed methods	**Online Medical Information Seeking Behaviour:** Patients explicitly asked if they used the internet to search for medical information<br>**Patient satisfaction:** A 29-statement scale[2]<br>**Recall of medical information:** A structured telephone interview using an adapted version of the Netherlands Patient Information Recall Questionnaire (NPIRQ)[1] (51)<br>**Medication adherence:** A single item self-report measure<br>**Discussion of online medical information:** Mention of (any combination of) the words *internet*, *Google(d)*, *webpages*, *fora*, *online*, or any other internet-related words during the recorded consultation and the person initiating the discussion	Yes - part of a larger research project developing and testing a theoretical and evidence- based tailored multimedia intervention to improve medication adherence in patients with IBD
(22)	How is online health information seeking discussed by patients and nurse practitioners?	Ulcerative colitis<br><br>Crohn's disease	**Patients** (Main study: n=165; current sub-study: n=58)<br>• Diagnosed with IBD<br>• Starting immunosuppressive and biological therapy<br>**IBD nurses** (n=8)	Netherlands	Mixed methods	**Communicative strategies used to discuss online health information:** Quantitative content analysis	Yes - part of a larger research project developing and testing a theoretical and evidence-based intervention to improve medication adherence in patients with IBD
(23)	How are the opportunities for the integration of patients' concerns enabled or hindered by the gastroenterologist?	Ulcerative colitis<br><br>Crohn's	Main study<br>**Physicians** (n=3), **Patients** (n=16)<br>Reported case study	UK	Qualitative	**Patient expectations of the future consultation and patient reflection after the consultation:** Episodic interviews<br>**Interaction:** the notion of *proto-story*	Yes – part of a project investigating how and when the concerns of IBD patients are included within the treatment

	(implied)	disease	• A woman patient (with her young daughter) with a registrar<br>• The same patient with a consultant<br>• A woman in her mid- 30s, with a long history of ulcerative colitis who had become a mother for the first time six months previously (with her baby) with the consultant				protocols of their doctors
(24)	• Is there alignment between patients and physicians in terms of the impact of disease on the patient's QoL? (implied)<br>• What is perceived to be treatment success in patients' and doctors' view? (implied)<br>• In which visits the physician asked patients about QoL?<br>• Which patients were experiencing QoL and emotional impacts due to their disease and, of these, which patients discussed these impacts during the visit?<br>• Which patients demonstrated evidence of resigning themselves to active symptoms as acceptable (i.e., 'a new normal' or 'learned helplessness')?<br>• In which visits the physician interrupted the patient and the number of interruptions?<br>• In which visits the physician asked primarily closed-ended questions to assess symptoms?<br>• In which visits the physician conveyed a	Ulcerative colitis	US study<br>**Gastroenterologists** (n=15)<br>• board-certified gastroenterologists, and spending at least 75% of their professional time in direct patient care<br>• be in full-time practice for 3 to 30 years,<br>• primarily see patients in an office or private practice setting<br>• see at least 25 patients diagnosed with UC in a typical month<br>• have initiated biologic therapy for at least 15% of their patients with UC<br>**Patients** (n=40)<br>• 18 years old or older<br>• Diagnosed with moderate-to-severe ulcerative colitis for ≥1 year<br>• At least one flare within the past year according to participating gastroenterologists<br>• Had previously received or were currently receiving therapy with 5-ASA and/or corticosteroids<br>• Fluent in English<br>• No cognitive impairment<br>EU study<br>**Gastroenterologists** (n= 8)	US and Europe (Italy, Germany, France)	Mixed methods	**Patient and physician attitudes toward and framing of immunomodulatory and biologic therapies:**<br>EU: Qualitative analysis of visits and post-visit interviews<br>US:<br>• Number of visits in which the physician conveyed a significant gap between conventional therapies and advanced therapies<br>• Number of visits in which the physician did not compare or discuss advance therapies<br>• Position of advanced therapies in the sequence of treatment in the visits in which they were discussed<br>• Number of visits in which gastroenterologists framed biologic therapy as a last resort<br>• Number of visits in which it was clear that gastroenterologists had previously discussed advanced options<br>• Number of patients who were not currently or previously prescribed advanced therapies and who discussed advanced therapy options during the post-visit interview and their appraisal of advanced therapies during the interview<br>**Patients and physicians' use and understanding of the term *inflammation***:<br>• Number of visits in which the term *inflammation* (or a variation thereof) was used by the physician<br>• Context in which the term *inflammation* was used by the physicians<br>• Number of visits in which the physician described what they meant by the term and explained the relevance of the term<br>• Number of visits in which the patient used the term *inflammation* (or variations)	No

	significant gap in their perception of conventional therapies for UC (e.g., 5-ASA and corticosteroids) and advanced therapies (e.g., immunomodulators and biologic therapies)?<br>• In which visits advanced therapies were framed as a last resort and thus considered only if absolutely necessary?<br>• In which visits advanced therapies were discussed and where they were positioned in the treatment sequence?<br>• Which patients considered advanced therapies as more appropriate for more severe stages of disease than they believed they were experiencing?		• Spending at least 60% of their time in direct patient care<br>• Primarily treating adults<br>• Main speciality being gastroenterology<br>• Planning to discuss immunosuppressant or biologic treatment with their patient at their next visit<br>• In full-time practice for 3 to 30 years<br>• Primarily seeing patients in an office or private practice setting<br>• Seeing at least 25 patients diagnosed with ulcerative colitis in a typical month<br>• Have initiated biologic therapy for at least 15% of their patients with ulcerative colitis<br>• **Patients** (n=28)<br>18 years old or older<br>• Diagnosed with moderate-to-severe ulcerative colitis for ≥1 year<br>• At least one flare within the past year according to participating gastroenterologists<br>• Had previously received or were currently receiving therapy with 5-ASA and/or corticosteroids<br>• No cognitive impairment			• Number of interviews in which the patient used the term *inflammation* and context in which the patients used the term *inflammation*<br>**Patient and physician description of treatment success:**<br>Analysed in terms of absence of clinical symptoms (clinical remission) or absence of inflammation (endoscopic remission)<br>**Further measures in the US study:**<br>• **Alignment between patients and physicians on QoL impacts:** (1) complete alignment; (2) partial alignment; (3) no alignment<br>• **Number of visits in which the physician asked patients about QoL or emotional impact of the disease**<br>• **Number of patients who were experiencing QoL and emotional impacts due to their disease and number of patients who did not discuss or underplay these impacts during the visit**<br>• **Number of patients who demonstrated evidence of resigning themselves to active symptoms as acceptable (i.e., 'a new normal' or 'learned helplessness')**<br>• **Percentage of words spoken by the participants and the length of each visit**<br>• **Number of visits in which the physician interrupted the patient and the number of interruptions**<br>• **Number of visits in which the physician asked primarily closed-ended questions to assess symptoms**	

1. Jansen J, van Weert J, van der Meulen N, van Dulmen S, Heeren T, Bensing J. Recall in older cancer patients: measuring memory for medical information. Gerontologist. 2008;48(2):149-57.
2. Linn AJ, van Weert JCM, van Dijk L, Horne R, Smit EG. The value of nurses' tailored communication when discussing medicines: Exploring the relationship between satisfaction, beliefs and adherence. Journal of Health Psychology. 2016;21(5):798-807.

Table 2. Characteristics of the interaction data

Paper	Year	Country	Setting	Number of sites	Corpus size	Average length of consultations	Whether series or one off	Participants	Patient demographics
(20)	2013	Netherlands	Nurse-patient prescribing consultation - Hospital	6	59 video-recorded and 9 audio-recorded consultations (68 consultations)	29.5 min (SD = 8.5)	One off	Nurses (n=8)<br>Patients (n=68)	Gender (N): Female: 42, Male: 26<br>Age M (SD): 40.5 (14.9)<br>Condition*: CD: 54, UC: 13, Unknown:1<br>Diagnosed in years M (SD): 9.6 (10.3)<br>Education level: Low: 18, Moderate: 24, High: 26<br>Living arrangements: Alone: 16, With partner: 16, With partner and child(ren) 16, With Child(ren): 8, Other: 12<br>Employed: yes: 51<br>Ethnicity: Dutch: 62
(21)	2018	Netherlands	Nurse-patient prescribing consultation - Hospital	6	165 video-recorded consultations (5 excluded from analysis due to lack of demographic data): 160 consultations	Not stated	One off	Nurses (n=8)<br>Patients (n=160)	Gender (N): Female: 92, Male: 68<br>Age M (SD): 43.10 (15.33)<br>Condition (N): CD: 101, UC: 49, other: 10<br>Diagnosed in years M (SD): 11.61 (10.55), Range: 1.5-47.1<br>Educational level: Low: 36, Moderate: 61, High: 63
(22)	2020	Netherlands	Nurse-patient prescribing consultation - Hospital	6	Main study: 165 recorded consultations<br>Current sub-study: 58 recorded consultations	Not stated	One off	Nurses (n=8)<br>Patients (n=58)	Gender (N): Female: 33, Male: 25<br>Age M: 48<br>Condition (N): CD: 34, UC: 20, Unknown: 2<br>Diagnosed in years M: 10<br>Educational level: Low: 6, Moderate: 22, High: 28, Unknown: 2
(23)	2008	UK	Outpatient clinic - Hospital	1	3x16 video-recorded consultations (assumed info, not explicitly stated in the article)	Not stated	Series (3 consultations from each patient)	Physicians (n=3)<br>Patients (n=16)	Not stated

For peer review only - http://bmjopen.bmj.com/site/about/guidelines.xhtml

(24)	2017	US and Europe (Italy, Germany, France)	outpatient clinics -community-based practices	Not stated	US study: 40 visits<br>EU study: 28 visits	15 min (U.S. research)	One off	US study.:<br>Gastroenterologists (n=15)<br>Patients (n=40)<br>EU study:<br>Gastroenterologists (n=8, Italy: 5, Germany: 2, France: 1)<br>Patients (n=28)	US study:<br>Gender: Female: 21, Male: 19<br>Age M (Range): 49 (20-83)<br>EU study:<br>Gender: Female: 11, Male: 17<br>Age:<br>18-34 years old: 4 patients<br>35-54 years old: 14 patients<br>55-74 years old: 4 patients<br>Unavailable: 6 patients

* CD: Crohn's disease, UC: ulcerative colitis

For peer review only

Figure Legends

Figure 1. The screening and inclusion process

Figure 2. Findings of articles investigating nurse-patient communication categorised by linguistic metafunction

Figure 3. Findings of articles investigating gastroenterologist-patient communication categorised by linguistic metafunction

For peer review only

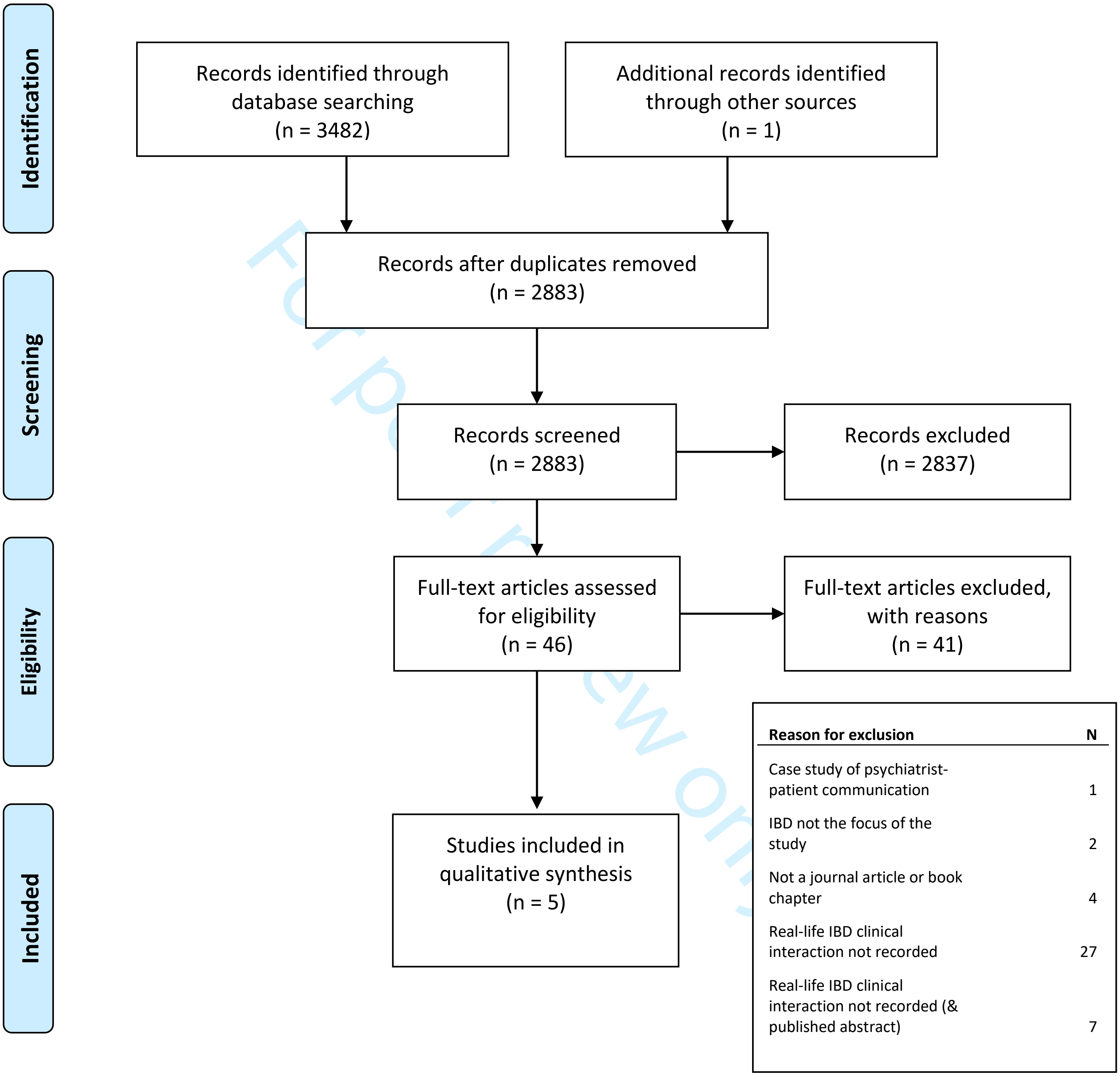

Figure 1. The screening and inclusion process

Interpersonal · Experiential · Textual

Nurse-patient clinical communication

(20)

Direct reference to online health information: In 46.8% of the 95 consultations (n=44), online health information was directly referenced. Overall, reference to online health information was almost evenly initiated by patients (n=33; 55%) and health-care providers (n=27; 45%).

Patients who directly refered to online health information during consultation were more satisfied with the consultation (mean=0.86; SD=.61) compared to patients who searched online but did not talk about it (mean=1.30; SD=1.13, Mdiff=-0.43, p=.041, d=0.48).

There was no significant relation between reference to online health information and recall of medical information (Mdiff=0.12, p=0.310).

There was no significant direct relation between patient satisfaction and the recall of medical information (Mdiff=-0.13, p=0.270)
For patients referring to online health information, higher satisfaction with the consultation related to higher recall of medical information, compared to not discussing it (Mdiff=0.32, p=0.054).

(21)

Medical information discussed (% of consultations in which each category was discussed): Purpose of the medication (89.7%); Name of medication (98.5%); Duration of treatment (48.5%); Frequency of administration (97.1%); Influence of the medication on the immune system (88.2%); When to expect an effect from the medication (80.9%); The need for blood monitoring (80.9%); Side effects (100%); When to contact the nurse (94.1%); The possibility of experiencing side effects (47.1%); The impact of the medication on the patients' daily life (79.4%); medication intake behaviour advice (44.1%)

Medical information recalled (%): Purpose of the medication (32.7%); Name of medication (83.6%); Duration of treatment (59.8%); Frequency administration (85.9%); Influence of the medication on the immune system (69.2%); When to expect an effect from the medication (66.8%); The need for blood monitoring (63.6%); Side effects (26.7%); When to contact the nurse (39.2%); The possibility of experiencing side effects (78.8%); The impact of the medication on the patients' daily life (5.5%); Medication intake behaviour advice (15%).

Delayed recall of medical information was significantly related to self-reported medication intake behavior (β=0.37, p=0.007). Patients with lower recall scores three weeks after the consultation rated themselves as less adherent than the patients with higher recall scores.

(22)

Discussions about online health information-seeking initiated

Patient's initiation strategies
Making a general statement (n=19, 43 %)
Raising concerns (n=8, 18 %)
Asking questions (n=7, 16 %)
Citing specific information (n=5, 11 %)
Dismissing (n=5, 11 %)
Nurses reaction strategies
Taking patients' online health information-seeking seriously (n=20, 43 %)
Affirming patients' beliefs (n=11, 24 %)
Ignoring (n=6, 13 %)
Minimal encouragement (n=5, 11 %)
Correcting (n=5, 11 %)

Discussions about online health information-seeking initiated by nurses:
Nurse's initiation strategies
Exploring (n=21, 50 %)
Warning (n= 13, 31 %)
Encouraging (n=8, 19 %)
Patient's reaction strategies
Disclosing (n=15, 40 %)
Reacting minimally (n=9, 24 %)
Expressing concerns (n=8, 21 %)
Substantive reaction (n=6, 16 %)

Nurse	Recorded consultations, n	Consultations in which the internet is discussed, n (%)	Nurse initiations, n (%)
1	17	4 (23%)	1 (25%)
2	10	1 (10%)	1 (100%)
3	16	3 (18%)	0 (0%)
4	15	6 (40%)	5 (83%)
5	15	8 (53%)	7 (88%)
6	50	20 (40%)	16 (80%)
7	32	14 (44%)	5 (36%)
8	25	1 (4%)	0 (0%)

Figure 2. Findings of articles investigating nurse-patient communication categorised by linguistic metafunction

Interpersonal | Ideational | Textual

Gastroenterologist-patient clinical communication

(24)

The way doctors sit, make references to their notes, ask questions, provide information, construct the concept of time and space, their posture, tone of voice, speed of talking, pauses, and interruptions mediate their view of the pateint's illness: medico-scientific view versus medicopresentational view.

Effect on patient experience: In the consultation in which the doctor held a medico-scientific view of the illness, the patient noted that the doctor "did not really listen to what she had to say".
In the consultation in which the doctor had a medicopresentational view of the illness, the patient noted that the doctor put her "on the right road really".

(23)

Question asking: Gastroenterologists typically used closed questions (73% of the visits, n=29) when assessing patients' symptoms and disease progression. Completion of electronic medical records often guided the dialogue and thus eye contact and elaboration of symptoms by the patient were limited.

Discussing QoL and emotional impact: Gastroenterologists asked patients about their QoL or emotional well-being in 23% (n=9) of visits. Fifty-five percent of the 31 patients who were experiencing QoL impacts either did not discuss these impacts during their visit (n=10; 32%) or underplayed the impacts (n=7; 23%). Of patients experiencing symptoms, most demonstrated evidence of resigning themselves to a 'new normal'.

Inflammation **:** The term *inflammation* (or a variation thereof) was used by gastroenterologists in 60% (n=24) of visits and an average of 4 times per visit. Gastroenterologists described what they meant by the term in only one (2.5%) of the visits and explained the relevance of the term in 3 (7.5%). Patients used the term *inflammation* (or variations) in 13% (n=5) of visits. During post-visit interviews, patients voluntarily used the term in 48% (n=19) of cases, but none described what they meant by the term and none demonstrated clinical understanding of it. Patients' use of the term was most frequently to justify treatment (30% of all visits; n=12) or in the assessment of their disease (15%; n=6).

Appraisal of advanced therapies: US gastroenterologists conveyed a significant risk level gap between conventional therapies and advanced therapies in 58% of visits (n=23). In 53% of visits (n=21), gastroenterologists framed biologic therapy as a last resort, to be considered only if absolutely necessary. The position of immunomodulatory in the treatment sequence was always undifferentiated.
EU gastroenterologist generally framed immunomodulatory therapies as an intermediate therapy for patients who had failed treatment with conventional therapies. Biologic therapy was positioned at the end of the treatment sequence, to be considered once other options had been considered and/or exhausted. Physicians positioned biologic agents as potent, effective therapies with known risks.

Goals of care: During visits with patients for whom the gastroenterologist viewed treatment success as endoscopic remission (10 visits), the gastroenterologist clearly communicated this goal in 40% of cases (n=4).

Attitudes toward treatment success: Gastroenterologists described treatment success for most patients as clinical remission (73% of cases; n=29). In 25% of cases (n=10), gastroenterologists described treatment success as endoscopic remission; in half of these 10 cases, endoscopic remission was an additional goal and in the other half, endoscopic remission was the only goal. Most patients (78%; n=31) described treatment success as an absence of clinical symptoms, i.e., clinical remission.

Interruptions: Gastroenterologists interrupted patients at least once in 63% (n=25) of visits. The average number of interruptions per visit was 1.5.

Contribution: Gastroenterologists spoke, on average, 61% of the words spoken during the visit and patients contributed 36% of the words spoken during visits.

Advanced therapies: Biologic therapies were not discussed in 40% of visits (n=16). Immunomodulatory therapy was discussed in 40% of visits (n=16). In 60% of visits (n=24), it was clear that gastroenterologists had previously discussed advanced options.

Patient attitude toward advanced therapies: Of the patients who were not currently or previously prescribed advanced therapies and who discussed advanced therapy options during the post-visit interview, all (n=17) considered advanced therapies as more appropriate for stages of disease more severe than their own.

EU gastroenterologists often structured the visit around a checklist of test results .
Fr US gastroenterologist, completion of electronic medical records often guided the dialogue and thus eye contact and elaboration of symptoms by the patient were limited.

The term *inflammation* was most frequently (n=20) used during the assessment of the patient's disease. Gastroenterologists also used the term during treatment justification (n=9) and disease education (n=8), among other contexts.

Physician-patient alignment (in terms of the asssessment of the QoL impacts): Complete alignment was demonstrated for 40% (n=16) of the consultations, partial alignment for 43% (n=17) of the consultation, and no alignment for 18% (n=7) of the consultations. Of the 17 consultations in which partial alignment was demonstrated, 12 were due to the gastroenterologist underestimating the QoL impacts; 3 were due to gastroenterologist overestimation; and 2 were due to the gastroenterologist and patient mentioning different QoL impacts.

Effect of physician interruption on the length of the visit: The average visit length increased with the number of interruptions.

For peer review only - http://bmjopen.bmj.com/site/about/guidelines.xhtml

Figure 3. Findings of articles investigating gastroenterologist-patient communication categorised by linguistic metafunction

Supplementary Table 1. Data items included in the data extraction template

- **Participant characteristics**
  - Participants and numbers
  - Participant socio-demographics
  - Additional health status information (e.g. pregnancy status, adult versus adolescent)
- **Disease characteristics**
  - Type of IBD
  - Disease activity
  - Disease phenotype
  - Extraintestinal manifestations
- **Study characteristics**
  - Year of research
  - Country of research
  - Research setting and type of consultation
  - Stated aims
  - Aims relevant to the review
  - Study design
  - Outcomes and measures
  - Stated findings
  - Stated conclusions
- **Consultation data and analysis characteristics**
  - Corpus size and number of consultations/episodes analysed in the study
  - Average length of consultations
  - One-off consultation or series
  - Data type (audio or video)
  - Method of linguistic data analysis (sociolinguistics, conversation analysis, content analysis, etc.)
  - Linguistic component/s analysed

For peer review only

Supplementary Table 2a. Analyses of risk of bias (tailored framework)

	Credibility									Reliability	
Article	Type of consultation data	Size of consultation data analysed	Sample size justification	Linguistic component(s) analysed	Linguistic data analysis tool(s)	Demographic data	Additional contextual information		Arguments/findings supported by textual data (1-3: 1=NO and 3 =YES)	Unit of analysis	Second coder
(20)	Nurse-patient consultation	95 video-recorded consultations (including patients who searched for medication-related online information prior to the consultation)	No	Lexis	Presence/absence of a list of words and the speaker	Yes	No		1	No	No
(21)	Nurse-patient consultation	59 video-recorded, 9 audio-recorded consultations (68 consultations)	No	Topic	An observation checklist (presence/absence)	Yes	No		1	No	Yes
(22)	Nurse-patient consultation	58 video- or audio-recorded consultations	No	Speech act	A data-driven codebook	Yes*	No		2	No	Yes
(23)	Gastroenterologist-patient consultation	2 video-recorded consultation	No	Sitting, Reference to notes, Representation of space and time	*Proto-story*	No	Yes	• Patient interviews<br>• Information on patients' personal life and consultant level of experience	2	No	No

For peer review only

(24)	Gastroenterologist-patient consultation	68 audio- and video-recorded consultations	No	Lexis, Topic, Question asking, Contribution to conversation, Interruptions, framing	Lexis: presence/absence, context and co-text, speaker<br>Topic: presence/absence, speaker<br>Question-asking: open-ended vs. closed<br>Contribution to conversation: Percentage of words spoken by the participants and the length of each visit<br>Interruptions: Number<br>Framing: Position in treatment sequence	Yes	Yes	• Patient and consultant interviews	2	No	No
* Inconsistent reports of data on IBD type: While the total sample size was reported to be 58, information on IBD type was reported for 56 participants (34 participants with Crohn's disease, 20 participants with ulcerative colitis, and two participants with unknown disease).											

Supplementary Table 2b. Analysis of risk of bias (Mixed Methods Appraisal Tool)

	**Mixed Methods Appraisal Tool (MMAT) – Version 2011**	**(20)**	**(21)**	**(22)**
Quantitative nonrandomized	Are participants (organizations) recruited in a way that minimizes selection bias?	No - 63.12% of participants had CD, 77.5% of the participants were moderately or highly educated	No - 62.3% of participants were female, 79.4% had CD, 73.5% were moderately or highly educated, 91.2% were Dutch	No – 35% of participants had UC, 87% of the participants were moderately or highly educated
	Are measurements appropriate (clear origin, or validity known, or standard instrument; and absence of contamination between groups when appropriate) regarding the exposure/intervention and outcomes?	Online Medical Information Seeking Behaviour: Patients explicitly asked if they used the internet to search for medical information ("yes" or "no") - **Yes**<br>Patient satisfaction: a 29-statement scale (Linn, van Weert, van Dijk, Horne, & Smit, 2016) – **Can't tell, tool could not be accessed**<br>Recall of medical information: a structured telephone interview using an adapted version of the Netherlands Patient Information Recall Questionnaire (NPIRQ) (Jansen et al., 2008) - **Yes**<br>Medication adherence: A single item self-report measure (Linn et al., 2013) - **Yes**	Immediate recall of medical information: An adapted version of The Netherlands Patient Information Recall Questionnaire (NPIRQ) - **Yes**<br>Delayed recall of medical information: An adapted version of the immediate recall questionnaire based on each individual consultation - **Yes (immediate and delayed recall measures were not the same)**<br>Percentage of accurate recall: The ratio of the number of the accurately recalled items to the total number of items discussed coded by two coders using videotaped visits - **Yes**<br>Total recall score: the mean recall percentage per patient for immediate	Yes - Communication strategies to Discuss Online Health Information: A data-driven codebook

For peer review only

			and delayed recall - **Yes**<br>Medication intake behaviour: A single item self-report measure ("Please indicate on a scale from 1 to 10 the extent to which you are taking the medication as prescribed)" - **Yes**	
	In the groups being compared (exposed vs. non-exposed; with intervention vs. without; cases vs. controls), are the participants comparable, or do researchers take into account (control for) the difference between these groups?	N/A	N/A	N/A
	Are there complete outcome data (80% or above), and, when applicable, an acceptable response rate (60% or above), or an acceptable follow-up rate for cohort studies (depending on the duration of follow-up)?	Yes	Yes	Yes
Qualitative	Are the sources of qualitative data (archives, documents, informants, observations) relevant to address the research question (objective)?	Yes; However, only those consultations in which internet-related words were included in the analysis.	Yes	Yes; However, only those consultations in which internet-related words were included in the analysis. Consultations in which there was an implicit reference to online health information were not included.
	Is the process for analyzing qualitative data relevant to address the research question (objective)?	Yes	Yes	Yes
	Is appropriate consideration given to how findings relate to the context, e.g., the setting, in which the data were collected?	No - Discussion of online health information was regarded as a dichotomy. How the online information was discussed was not studied.	No - the effect of contextual factors was not considered in analysing the content of consultations.	No – While the co-text of communicative strategies was discussed to some degree, the effect of contextual factors was not considered in the analysis.
	Is appropriate consideration given to how findings relate to researchers' influence, e.g., through their interactions with participants?	Yes	The likely influence of video-recording consultation on participants' behaviour was not addressed.	Yes
Mixed methods	Is the mixed methods research design relevant to address the qualitative and quantitative research questions (or objectives), or the qualitative and quantitative aspects of the mixed methods question (or objective)?	Yes	Yes	Yes
	Is the integration of qualitative and quantitative data (or results*) relevant to address the research question (objective)?	Yes	Yes	Yes

For peer review only

	Is appropriate consideration given to the limitations associated with this integration, e.g., the divergence of qualitative and quantitative data (or results*) in a triangulation design?	N/A	N/A	N/A
Screening questions	Are there clear qualitative and quantitative research questions (or objectives*), or a clear mixed methods question (or objective*)?	Yes	Yes	Yes
	Do the collected data allow address the research question (objective)? E.g., consider whether the follow-up period is long enough for the outcome to occur (for longitudinal studies or study components).	Yes	Yes	Yes

* CD: Crohn's disease, UC: ulcerative colitis

For peer review only

PRISMA 2009 Checklist

Section/topic	#	Checklist item	Reported on page #
**TITLE**			
Title	1	Identify the report as a systematic review, meta-analysis, or both.	1
**ABSTRACT**			
Structured summary	2	Provide a structured summary including, as applicable: background; objectives; data sources; study eligibility criteria, participants, and interventions; study appraisal and synthesis methods; results; limitations; conclusions and implications of key findings; systematic review registration number.	2
**INTRODUCTION**			
Rationale	3	Describe the rationale for the review in the context of what is already known.	3
Objectives	4	Provide an explicit statement of questions being addressed with reference to participants, interventions, comparisons, outcomes, and study design (PICOS).	3
**METHODS**			
Protocol and registration	5	Indicate if a review protocol exists, if and where it can be accessed (e.g., Web address), and, if available, provide registration information including registration number.	3
Eligibility criteria	6	Specify study characteristics (e.g., PICOS, length of follow-up) and report characteristics (e.g., years considered, language, publication status) used as criteria for eligibility, giving rationale.	3-4
Information sources	7	Describe all information sources (e.g., databases with dates of coverage, contact with study authors to identify additional studies) in the search and date last searched.	4
Search	8	Present full electronic search strategy for at least one database, including any limits used, such that it could be repeated.	4
Study selection	9	State the process for selecting studies (i.e., screening, eligibility, included in systematic review, and, if applicable, included in the meta-analysis).	4
Data collection process	10	Describe method of data extraction from reports (e.g., piloted forms, independently, in duplicate) and any processes for obtaining and confirming data from investigators.	4
Data items	11	List and define all variables for which data were sought (e.g., PICOS, funding sources) and any assumptions and simplifications made.	4
Risk of bias in individual studies	12	Describe methods used for assessing risk of bias of individual studies (including specification of whether this was done at the study or outcome level), and how this information is to be used in any data synthesis.	4
Summary measures	13	State the principal summary measures (e.g., risk ratio, difference in means).	N/A
Synthesis of results	14	Describe the methods of handling data and combining results of studies, if done, including measures of consistency (e.g., I^2) for each meta-analysis.	4

For peer review only - http://bmjopen.bmj.com/site/about/guidelines.xhtml

PRISMA 2009 Checklist

Section/topic	#	Checklist item	Reported on page #
Risk of bias across studies	15	Specify any assessment of risk of bias that may affect the cumulative evidence (e.g., publication bias, selective reporting within studies).	N/A
Additional analyses	16	Describe methods of additional analyses (e.g., sensitivity or subgroup analyses, meta-regression), if done, indicating which were pre-specified.	4
**RESULTS**			
Study selection	17	Give numbers of studies screened, assessed for eligibility, and included in the review, with reasons for exclusions at each stage, ideally with a flow diagram.	5
Study characteristics	18	For each study, present characteristics for which data were extracted (e.g., study size, PICOS, follow-up period) and provide the citations.	5
Risk of bias within studies	19	Present data on risk of bias of each study and, if available, any outcome level assessment (see item 12).	5
Results of individual studies	20	For all outcomes considered (benefits or harms), present, for each study: (a) simple summary data for each intervention group (b) effect estimates and confidence intervals, ideally with a forest plot.	6 and figures 2 & 3
Synthesis of results	21	Present results of each meta-analysis done, including confidence intervals and measures of consistency.	N/A
Risk of bias across studies	22	Present results of any assessment of risk of bias across studies (see Item 15).	N/A
Additional analysis	23	Give results of additional analyses, if done (e.g., sensitivity or subgroup analyses, meta-regression [see Item 16]).	6 and figures 2 & 3
**DISCUSSION**			
Summary of evidence	24	Summarize the main findings including the strength of evidence for each main outcome; consider their relevance to key groups (e.g., healthcare providers, users, and policy makers).	6-10
Limitations	25	Discuss limitations at study and outcome level (e.g., risk of bias), and at review-level (e.g., incomplete retrieval of identified research, reporting bias).	10
Conclusions	26	Provide a general interpretation of the results in the context of other evidence, and implications for future research.	10
**FUNDING**			
Funding	27	Describe sources of funding for the systematic review and other support (e.g., supply of data); role of funders for the systematic review.	1

From: Moher D, Liberati A, Tetzlaff J, Altman DG, The PRISMA Group (2009). Preferred Reporting Items for Systematic Reviews and Meta-Analyses: The PRISMA Statement. PLoS Med 6(7): e1000097. doi:10.1371/journal.pmed1000097

For more information, visit: **www.prisma-statement.org**.

For peer review only - http://bmjopen.bmj.com/site/about/guidelines.xhtml
